# Supplementary material for: Engineering of extracellular vesicles for efficient intracellular delivery of multimodal therapeutics including genome editors
Source: Nat Commun. 2025 Apr 29;16:4028. doi: 10.1038/s41467-025-59377-y (PMC12041237; doi:10.1038/s41467-025-59377-y)

Engineering of extracellular vesicles for efficient intracellular delivery of multimodal therapeutics including genome editors

Supplementary data

Supplementary figures

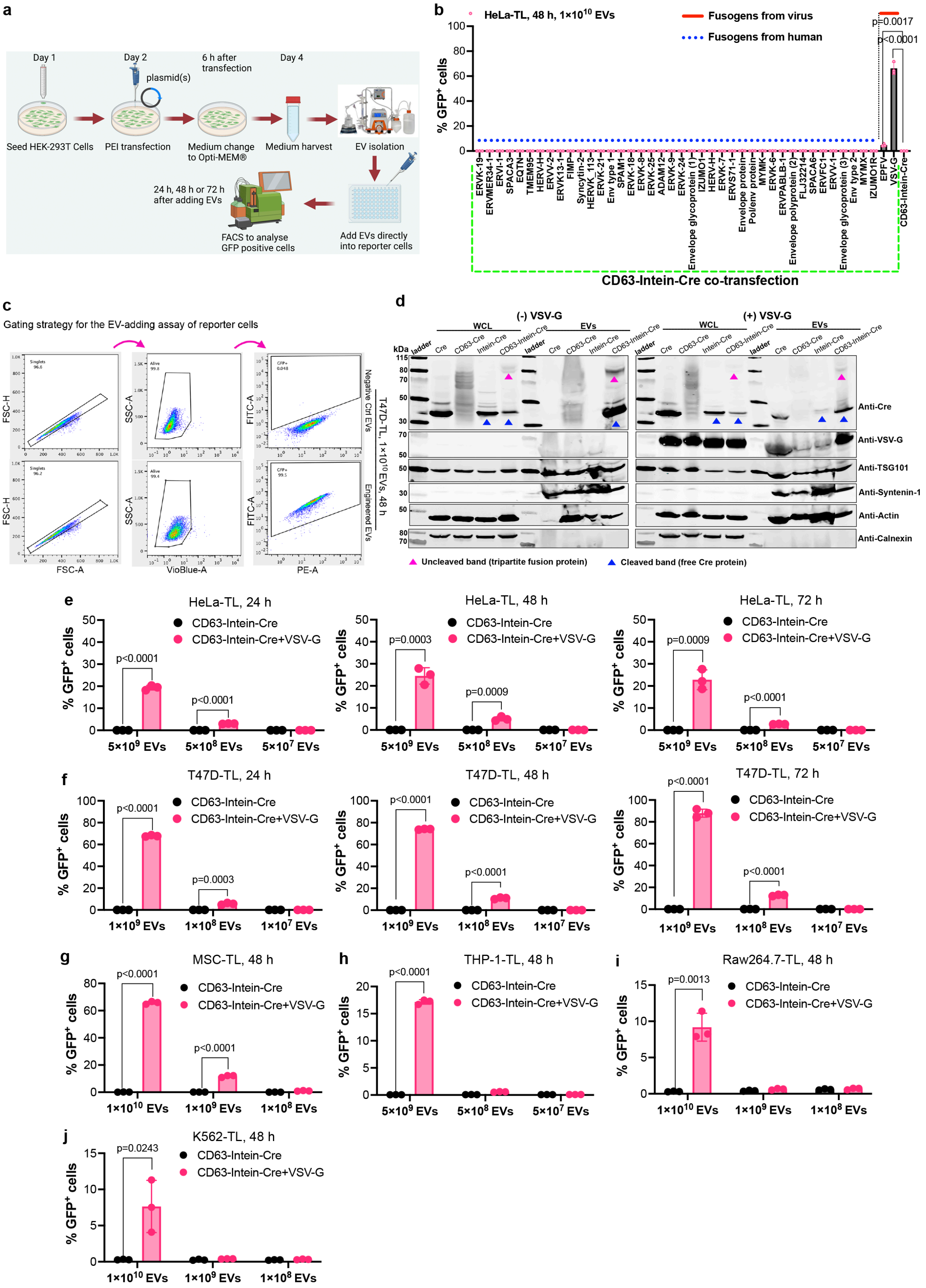

Supplementary Fig. 1. Development of VEDIC system for efficient intracellular protein delivery. a, A workflow schematic for the evaluation of EV-mediated Cre delivery to Cre recombinase fluorescent Traffic Light (TL) reporter cells and the subsequent analysis of GFP positive cells in recipient cells after adding isolated EVs. **b,** Fusogen screen in HeLa-TL cells after a two-day incubation with EVs. **c,** Example of the applied flow cytometry gating strategy for the analysis of GFP positive cells to evaluate Cre recombination after adding isolated EVs to fluorescent TL reporter cells. **d,** Protein expression of various engineered Cre fusion constructs in whole cell lysates (WCL) and isolated EVs derived from HEK293T cells evaluated by western blot (WB) analysis. Lysates from 5×10^5 EV-producing cells and 1×10^{10} engineered vesicles were used for the assay. TSG101, syntenin-1 and β-actin were used as EV markers and Calnexin was used as a cellular organelle marker (endoplasmic reticulum) and should be absent for EV samples. **e**,**f**, Percentage of GFP positive cells in HeLa-TL and T47D-TL reporter cells respectively after adding different doses of isolated engineered EVs for 1 to 3 days. (**g-j**) Percentage of recombined GFP positive cells mediated by intracellular Cre delivery through different doses of engineered EVs for 48 hours (h) in hard-to-transfect cells (**g:** MSC-TL; **h:** THP-1-TL; **i:** Raw264.7-TL; **j:** K562-TL). One-way ANOVA (Tukey) multiple comparisons test was used for analysis of (**b**); Two-way (Tukey) ANOVA multiple comparisons test was used for analysis of (**e-j**). **a** Created in BioRender.com, Zheng, W. (2025) *https://BioRender.com/s90c171*. Experiments were done with 3 biological replicates and data are shown as mean±SD. Exact p values ($p < 0.0001$) were reported in the Source Data. Source data are provided as a Source Data file.

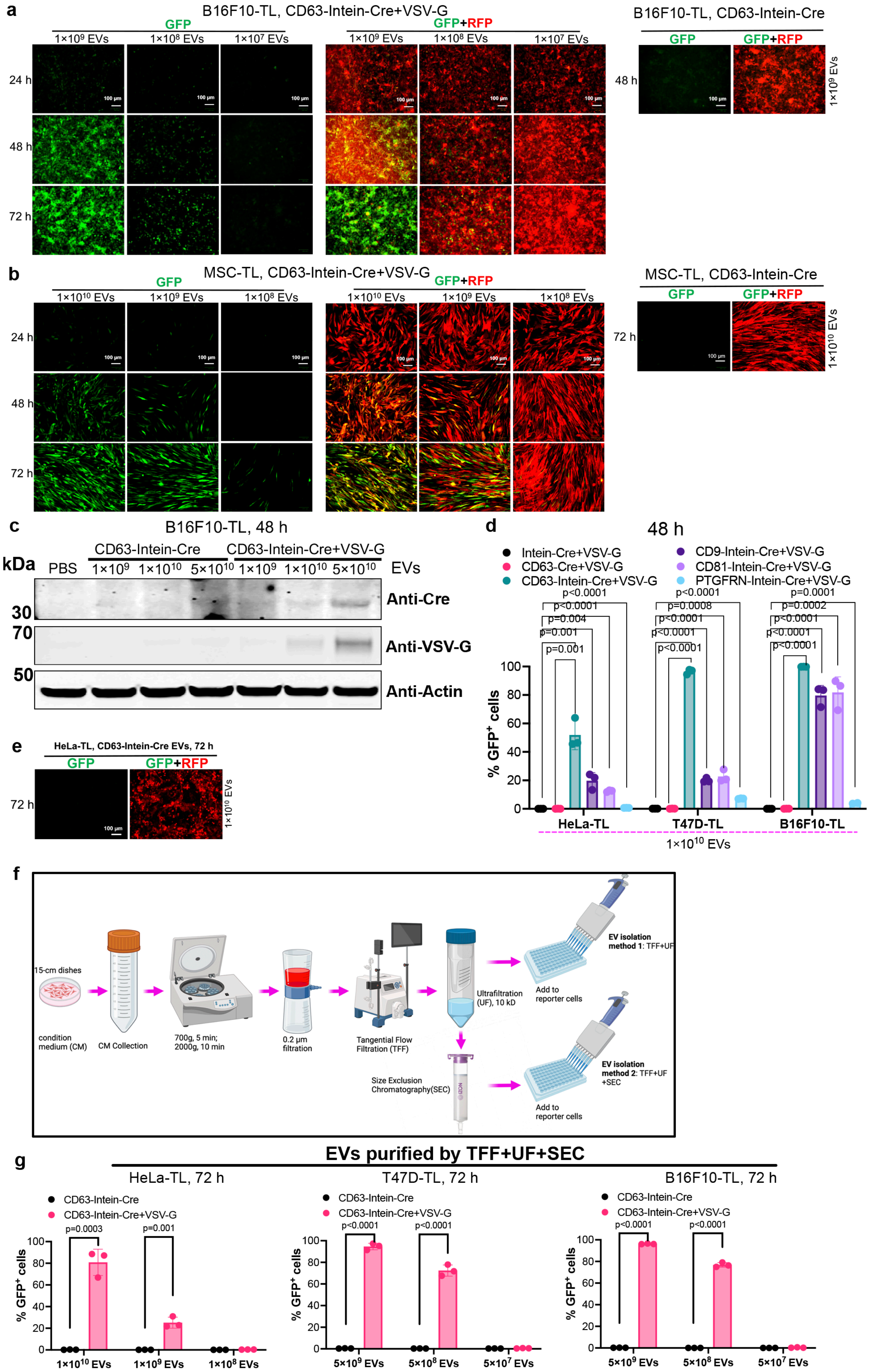
a
B16F10-TL, CD63-Intein-Cre+VSV-G
GFP
GFP+RFP
1×10⁹ EVs
1×10⁸ EVs
1×10⁷ EVs
24 h
48 h
72 h
100 µm
B16F10-TL, CD63-Intein-Cre
1×10⁹ EVs
b
MSC-TL, CD63-Intein-Cre+VSV-G
1×10¹⁰ EVs
1×10⁹ EVs
1×10⁸ EVs
MSC-TL, CD63-Intein-Cre
1×10¹⁰ EVs
c
B16F10-TL, 48 h
kDa
PBS
CD63-Intein-Cre
CD63-Intein-Cre+VSV-G
1×10⁹ 1×10¹⁰ 5×10¹⁰ 1×10⁹ 1×10¹⁰ 5×10¹⁰
EVs
30
70
50
Anti-Cre
Anti-VSV-G
Anti-Actin
d
48 h
Intein-Cre+VSV-G
CD63-Cre+VSV-G
CD63-Intein-Cre+VSV-G
CD9-Intein-Cre+VSV-G
CD81-Intein-Cre+VSV-G
PTGFRN-Intein-Cre+VSV-G
% GFP⁺ cells
HeLa-TL
T47D-TL
B16F10-TL
1×10¹⁰ EVs
e
HeLa-TL, CD63-Intein-Cre EVs, 72 h
1×10¹⁰ EVs
f
15-cm dishes
condition medium (CM)
CM Collection
700g, 5 min; 2000g, 10 min
0.2 µm filtration
Tangential Flow Filtration (TFF)
Ultrafiltration (UF), 10 kD
Size Exclusion Chromatography(SEC)
Add to reporter cells
EV isolation method 1: TFF+UF
EV isolation method 2: TFF+UF +SEC
g
EVs purified by TFF+UF+SEC
HeLa-TL, 72 h
T47D-TL, 72 h
B16F10-TL, 72 h
CD63-Intein-Cre
CD63-Intein-Cre+VSV-G
% GFP⁺ cells
1×10¹⁰ EVs
1×10⁹ EVs
1×10⁸ EVs
5×10⁹ EVs
5×10⁸ EVs
5×10⁷ EVs

Supplementary Fig. 2. Fluorescent microscopy Analysis of EV-mediated Cre delivery and test of different EV-sorting domains for VEDIC system. a,b, Fluorescent microscopy analysis of Cre-mediated B16F10-TL and MSC-TL reporter cell activation after adding indicated doses of EVs for indicated time of incubation. Scale bar, 100 µm, representative images. **c,** Cre and VSV-G proteins measured by WB in B16F10-TL cells after adding the indicated doses of engineered EVs in 24-well plates. **d,** Recombination in reporter cells mediated by EVs derived from engineered cells using different EV-sorting domains. **e,** Representative images showing the GFP positive HeLa-TL cells after adding EVs for 72 for CD63-Intein-Cre group (without VSV-G as negative control). Scale bar, 100 µm. **f,** Schematic illustration for the methods used to isolate engineered EVs in this study. **g,** The percentage of recombined reporter cells using VFIC EVs isolated by TFF+UF+SEC method. Two-way ANOVA (Tukey) multiple comparisons test was used for analysis of (**d**) and (**g**). **f** Created in BioRender.com, Zheng, W. (2025) *https://BioRender.com/v11h404*. Experiments were done with 3 biological replicates and data a are shown as mean±SD. Exact p values ($p < 0.0001$) were reported in the Source Data. Source data are provided as a Source Data file.

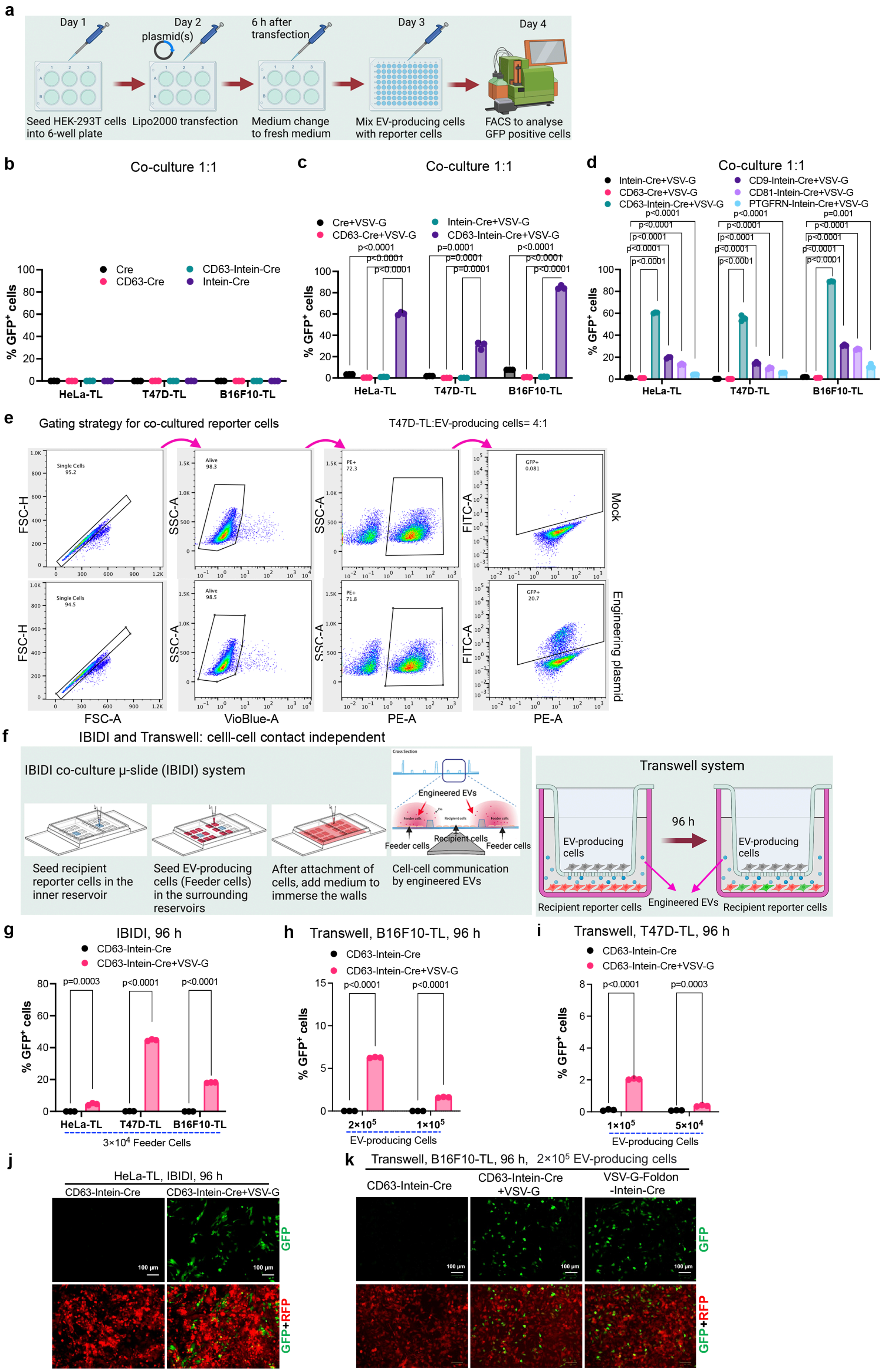

a
Day 1
Day 2
plasmid(s)
6 h after transfection
Day 3
Day 4
Seed HEK-293T cells into 6-well plate
Lipo2000 transfection
Medium change to fresh medium
Mix EV-producing cells with reporter cells
FACS to analyse GFP positive cells
b
Co-culture 1:1
Cre
CD63-Cre
CD63-Intein-Cre
Intein-Cre
% GFP⁺ cells
HeLa-TL
T47D-TL
B16F10-TL
c
Co-culture 1:1
Cre+VSV-G
CD63-Cre+VSV-G
Intein-Cre+VSV-G
CD63-Intein-Cre+VSV-G
p<0.0001
% GFP⁺ cells
HeLa-TL
T47D-TL
B16F10-TL
d
Co-culture 1:1
Intein-Cre+VSV-G
CD63-Cre+VSV-G
CD63-Intein-Cre+VSV-G
CD9-Intein-Cre+VSV-G
CD81-Intein-Cre+VSV-G
PTGFRN-Intein-Cre+VSV-G
p<0.0001
p=0.001
% GFP⁺ cells
HeLa-TL
T47D-TL
B16F10-TL
e
Gating strategy for co-cultured reporter cells
T47D-TL:EV-producing cells= 4:1
Single Cells 95.2
Alive 98.3
PE+ 72.3
GFP+ 0.081
Single Cells 94.5
Alive 98.5
PE+ 71.8
GFP+ 20.7
FSC-H
SSC-A
FITC-A
FSC-A
VioBlue-A
PE-A
Mock
Engineering plasmid
f
IBIDI and Transwell: celll-cell contact independent
IBIDI co-culture μ-slide (IBIDI) system
Engineered EVs
Feeder cells
Recipient cells
Seed recipient reporter cells in the inner reservoir
Seed EV-producing cells (Feeder cells) in the surrounding reservoirs
After attachment of cells, add medium to immerse the walls
Cell-cell communication by engineered EVs
Transwell system
96 h
EV-producing cells
Recipient reporter cells
Engineered EVs
g
IBIDI, 96 h
CD63-Intein-Cre
CD63-Intein-Cre+VSV-G
p=0.0003
p<0.0001
% GFP⁺ cells
HeLa-TL
T47D-TL
B16F10-TL
3×10⁴ Feeder Cells
h
Transwell, B16F10-TL, 96 h
CD63-Intein-Cre
CD63-Intein-Cre+VSV-G
p<0.0001
% GFP⁺ cells
2×10⁵
1×10⁵
EV-producing Cells
i
Transwell, T47D-TL, 96 h
CD63-Intein-Cre
CD63-Intein-Cre+VSV-G
p<0.0001
p=0.0003
% GFP⁺ cells
1×10⁵
5×10⁴
EV-producing Cells
j
HeLa-TL, IBIDI, 96 h
CD63-Intein-Cre
CD63-Intein-Cre+VSV-G
100 μm
GFP
GFP+RFP
k
Transwell, B16F10-TL, 96 h, 2×10⁵ EV-producing cells
CD63-Intein-Cre
CD63-Intein-Cre +VSV-G
VSV-G-Foldon -Intein-Cre
100 μm
GFP
GFP+RFP

Supplementary Fig. 3. Co-culture assays for validation of the VEDIC system for efficient intracellular protein delivery. a, Brief workflow for the direct co-culture assay of EV-producing cells and reporter cells to show EV-mediated Cre delivery. **b,** Co-culture assay for evaluation of Cre transfer without expression of VSV-G. **c,** Analysis of Cre-mediated recombination in cells after a co-culture assay with expression of VSV-G. **d,** Comparison of Cre transfer efficiency using different EV loading domains in a direct co-culture assay. **e,** Gating strategy utilized for the analysis of Cre recombination, as indicated by the percentage of GFP positive cells, in co-culture assay. **f,** Schematic graph to show the principle and workflow of IBIDI co-culture μ-slide (IBIDI) and Transwell co-culture assays demonstrating cell-cell contact independent Cre delivery from donor cells to recipient cells. The pore size for Transwell assay was 0.4 μm. **g,** Flow cytometry analysis of Cre delivery mediated by engineered EVs in a contact-independent co-culture assay using IBIDI assay. 3×10^4 EV-producing cells and 4×10^4 reporter cells were seeded into the surrounding reservoirs and central reservoir respectively and the analysis was performed after 4 days. **h**,**i**, Flow cytometry analysis of Engineered EV-mediated Cre delivery from donor cells to B16F10-TL and T47D-TL reporter cells respectively in a co-culture Transwell assay in 24-well plates. Indicated numbers of EV-producing cells were seeded into the upper chamber and the numbers of B16F10-TL and T47D-TL reporter cells in the lower chamber were 5×10^4 and 8×10^4 respectively in 24-well plate. Flow cytometry analysis was performed after 96 h. **j,** Representative fluorescence microscopy images showing Cre recombinase-induced GFP expression in HeLa-TL cells by IBIDI assay. Scale bar, 100 μm. **k,** Fluorescence microscopy images demonstrating GFP positive cells in B16F10-TL after a Transwell assay in 24-well plates. Scale bar, 100 μm, representative images. Two-way ANOVA (Tukey) multiple comparisons test was used for analysis of (**b**-**d**) and (**g**-**i**). **a, f** Created in BioRender.com, Zheng, W. (2025) *https://BioRender.com/p83s805,* Zheng, W. (2025) *https://BioRender.com/a83q292* and Zheng, W. (2025) *https://BioRender.com/r44d413* respectively. Experiments were done with 3 biological replicates and data were shown as mean±SD. Exact p values ($p < 0.0001$) were reported in the Source Data. Source data are provided as a Source Data file.

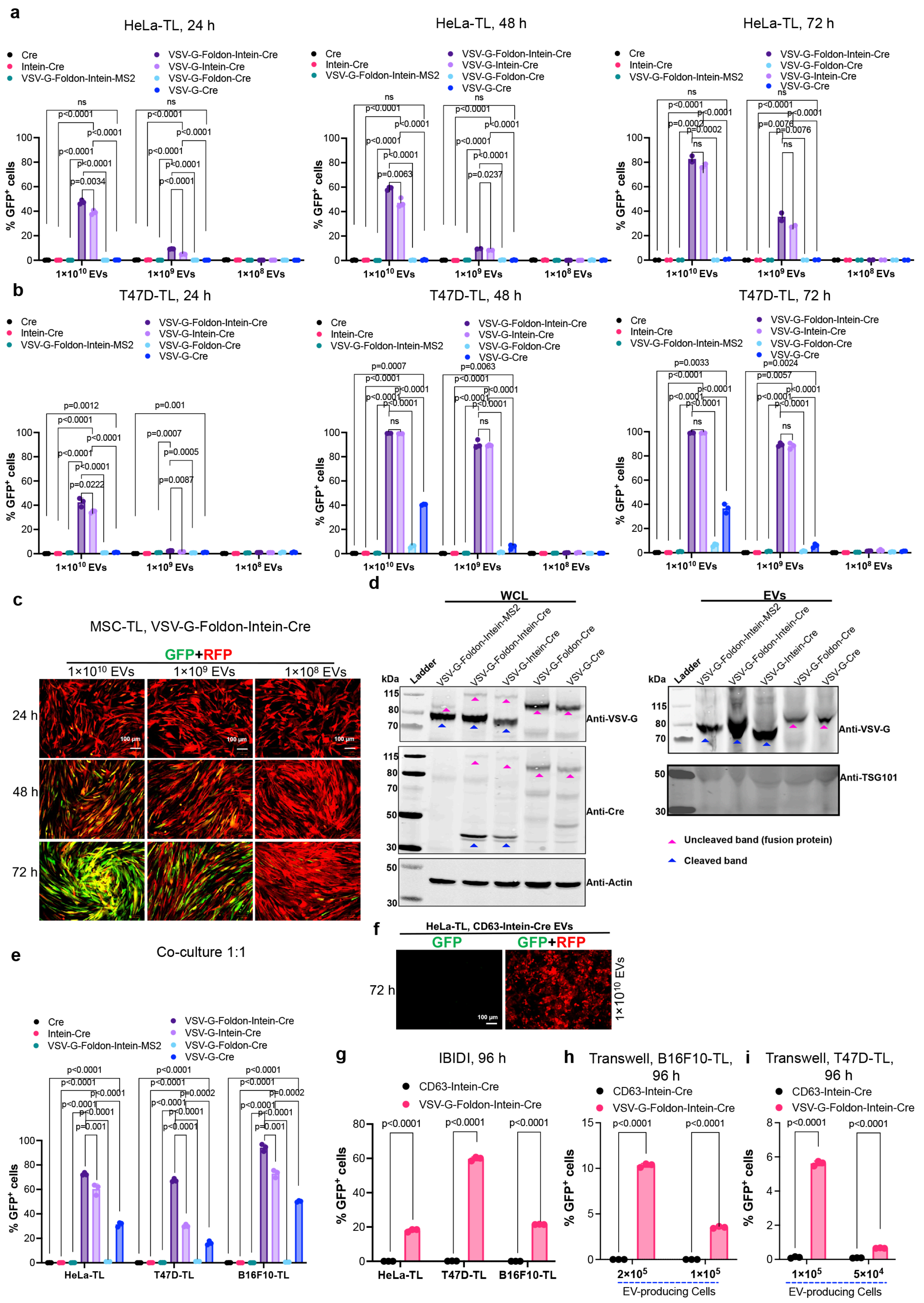

Supplementary Fig. 4. Development of VFIC system for efficient intracellular protein delivery. a, Percentage of GFP positive cells in HeLa-TL cells after adding engineered EVs for 1-3 days. Indicated doses of EVs were added. **b,** Percentage of recombined cells mediated by Cre from adding engineered EVs in T47D-TL cells for 1-3 days. **c,** Representative

fluorescence microscopy images showed the GFP expression in MSC-TL cells after EV-mediated Cre delivery. Indicated doses of isolated EVs were incubated for the indicated time. Isolated EVs from VFIC system demonstrated dose- and time-dependent Cre delivery into recipient cells. Scale bar, 100 µm. **d,** The expression of constructs related to VFIC system as measured by WB analysis. Proteins from 5×10^5 EV-producing cells and 1×10^{10} engineered vesicles were analyzed. TSG101 was included as EV marker. **e,** Flow cytometry analysis of a direct co-culture assay using various EV engineering strategies to assay Cre transfer after 24 h. **f,** Representative images demonstrating GFP positive HeLa-TL cells after adding CD63-Intein-Cre EVs for 72 h, negative control group for VSV-G-Foldon-Intein-Cre. Scale bar, 100 µm. **g,** Percentage of GFP positive cells evaluated by IBIDI assay for 96 h. 4×10^4 EV producing cells and 4×10^4 reporter cells were seeded into the surrounding reservoirs and central reservoir respectively and flow cytometry was performed after 4 days. **h**,**i,** Percentage of GFP positive cells in B16F10-TL and T47D-TL cells respectively evaluated by Transwell assay for 96 h. Indicated numbers of EV-producing cells were added into the up chamber of the Transwell system and co-cultured with 5×10^4 B16F10-TL or 8×10^4 T47D-TL reporter cells in the lower chamber in 24-well plates. Two-way ANOVA (Tukey) multiple comparisons test was used for analysis of (**a** and **b**) and (**e**,**g-i**). Experiments were done with 3 biological replicates and data are shown as mean±SD. Exact p values ($p < 0.0001$) were reported in the Source Data. Source data are provided as a Source Data file.

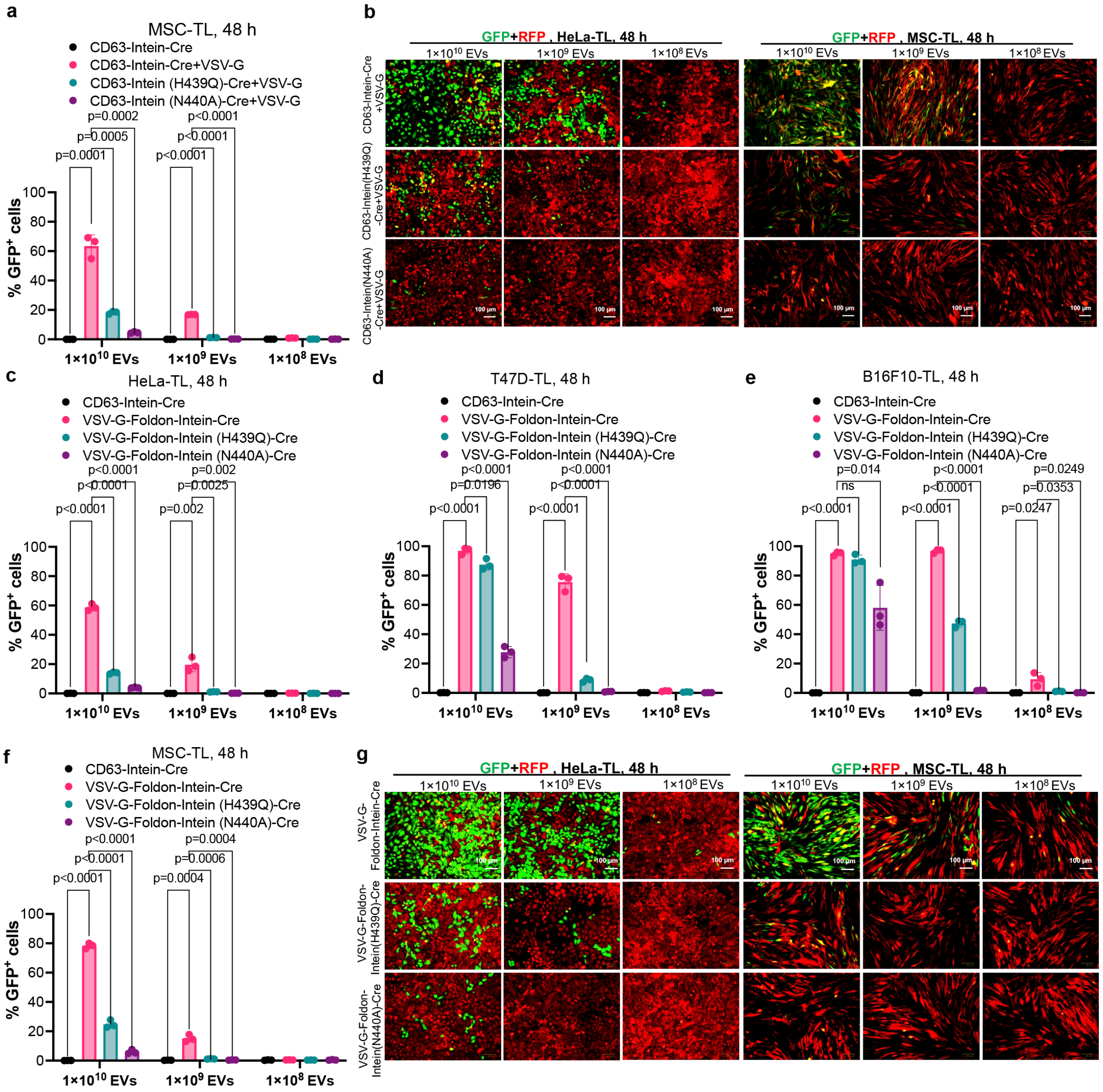

Supplementary Fig. 5. The pH-sensitive intein performs C-terminal cleavage during EV-biogenesis. a, Recombination in MSC-TL reporter cells mediated by EVs derived from engineered cells using mutant inteins (H439Q and N440A) in VEDIC system. **b,** Fluorescent images showing the GFP positive cells in Hela-TL and MSC-TL cells. Scale bar, 100 μm, representative images. **c-f,** Recombination in reporter cells (**c:** HeLa-TL; **d:** T47D-TL; **e:** B16F10-TL; **f:** MSC-TL) mediated by EVs derived from engineered cells using mutant inteins (H439Q and N440A) in VFIC system detected by flow cytometry. **g,** Representative fluorescent images for the GFP positive cells after 48 hours' adding various mutant intein variants engineered EVs at different doses for VFIC system. Scale bar, 100 μm. Two-way ANOVA multiple comparisons test was used for analysis of (**a**) and (**c-f**). Experiments were done with 3 biological replicates and data are shown as mean±SD. Exact p values ($p < 0.0001$) were reported in the Source Data. Source data are provided as a Source Data file.

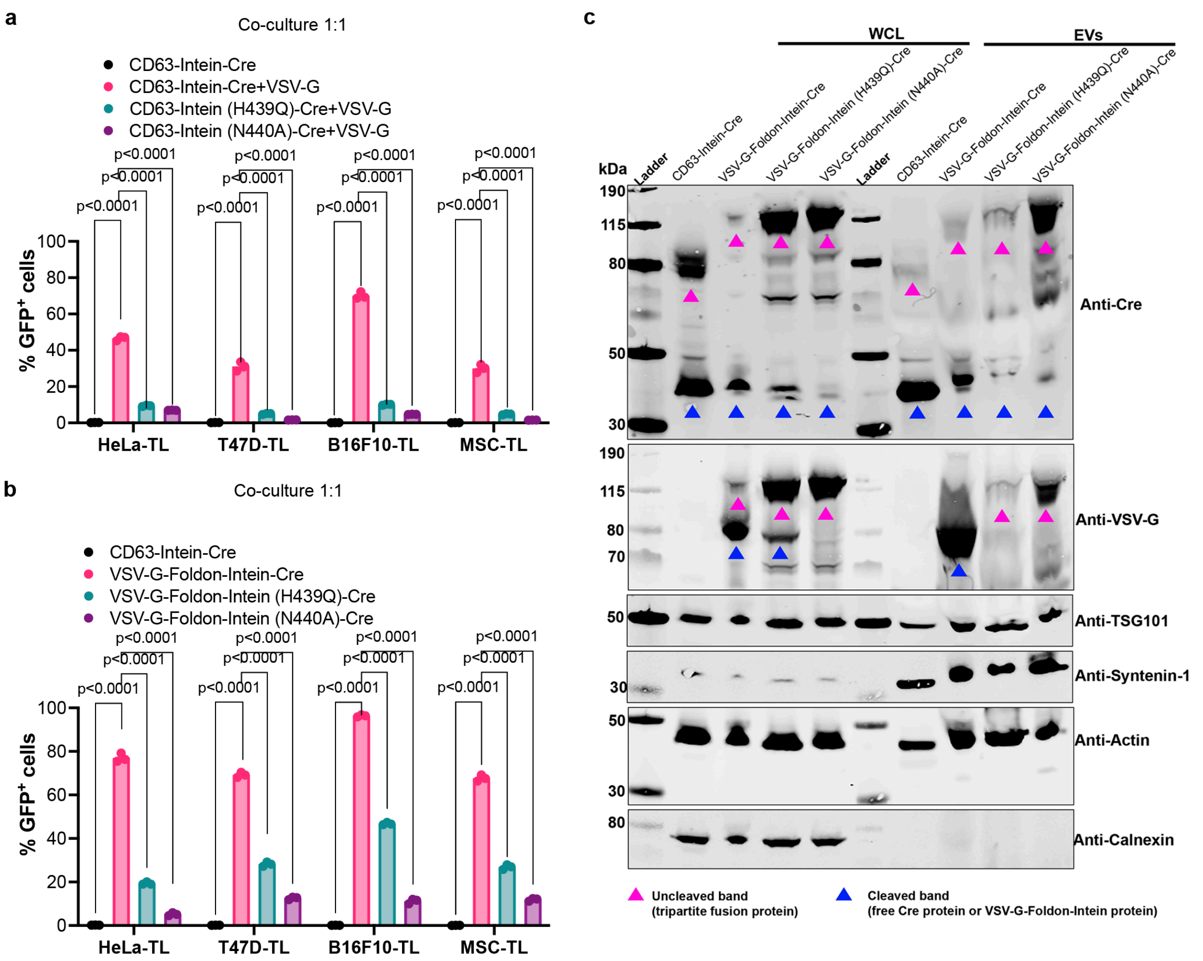

Supplementary Fig. 6. The pH-sensitive intein performs C-terminal cleavage during EV-biogenesis. a, Comparison of Cre transfer efficiency using different intein variants in a direct co-culture assay for VEDIC system. **b,** Flow cytometry analysis of a direct co-culture assay using intein variants to decrease Cre transfer for VFIC system. Co-culture was analyzed after 24 h incubation of the EV-producing cells and reporter cells. **c,** Protein expression of various engineered mutant intein constructs in WCL and isolated EVs derived from HEK-293T cells evaluated by WB analysis. Lysates from 5×10^5 EV-producing cells and 1×10^{10} engineered vesicles were used for the assay. TSG101, syntenin-1 and β-actin were used as EV markers and Calnexin was used as a cellular organelle marker (endoplasmic reticulum) and should be absent for EV samples. Two-way ANOVA (Tukey) multiple comparisons test was used for analysis of (**a** and **b**). Experiments were done with 3 biological replicates and data are shown as mean±SD. Exact p values ($p < 0.0001$) were reported in the Source Data. Source data are provided as a Source Data file.

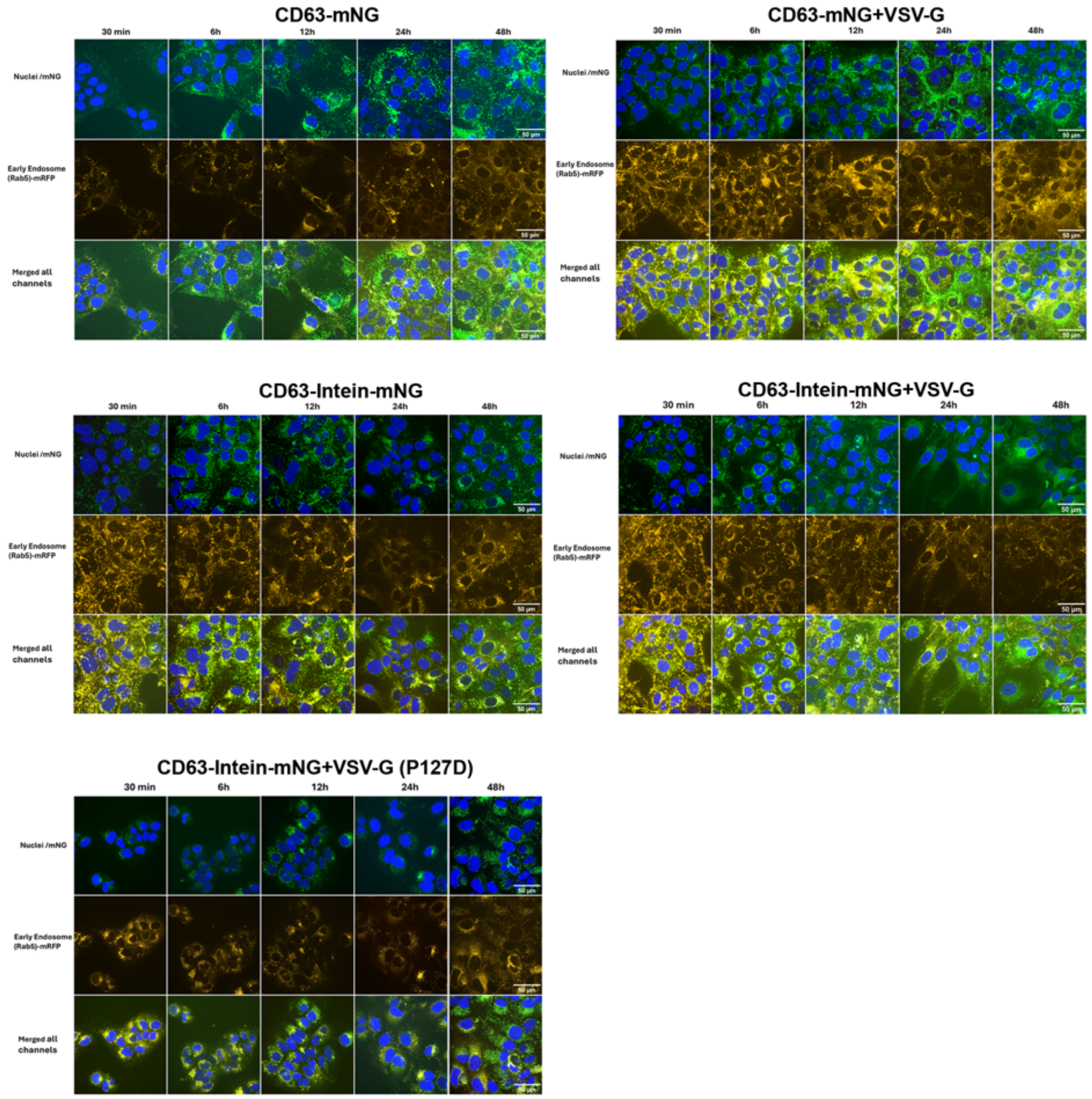

Supplementary Fig. 7. Co-localization of early endosome marker (Rab5) with the mNG in Huh7 cells at different time points (dynamic change) after adding engineered EVs. Rab5 was co-localized with mNG in different engineering groups at different time points.

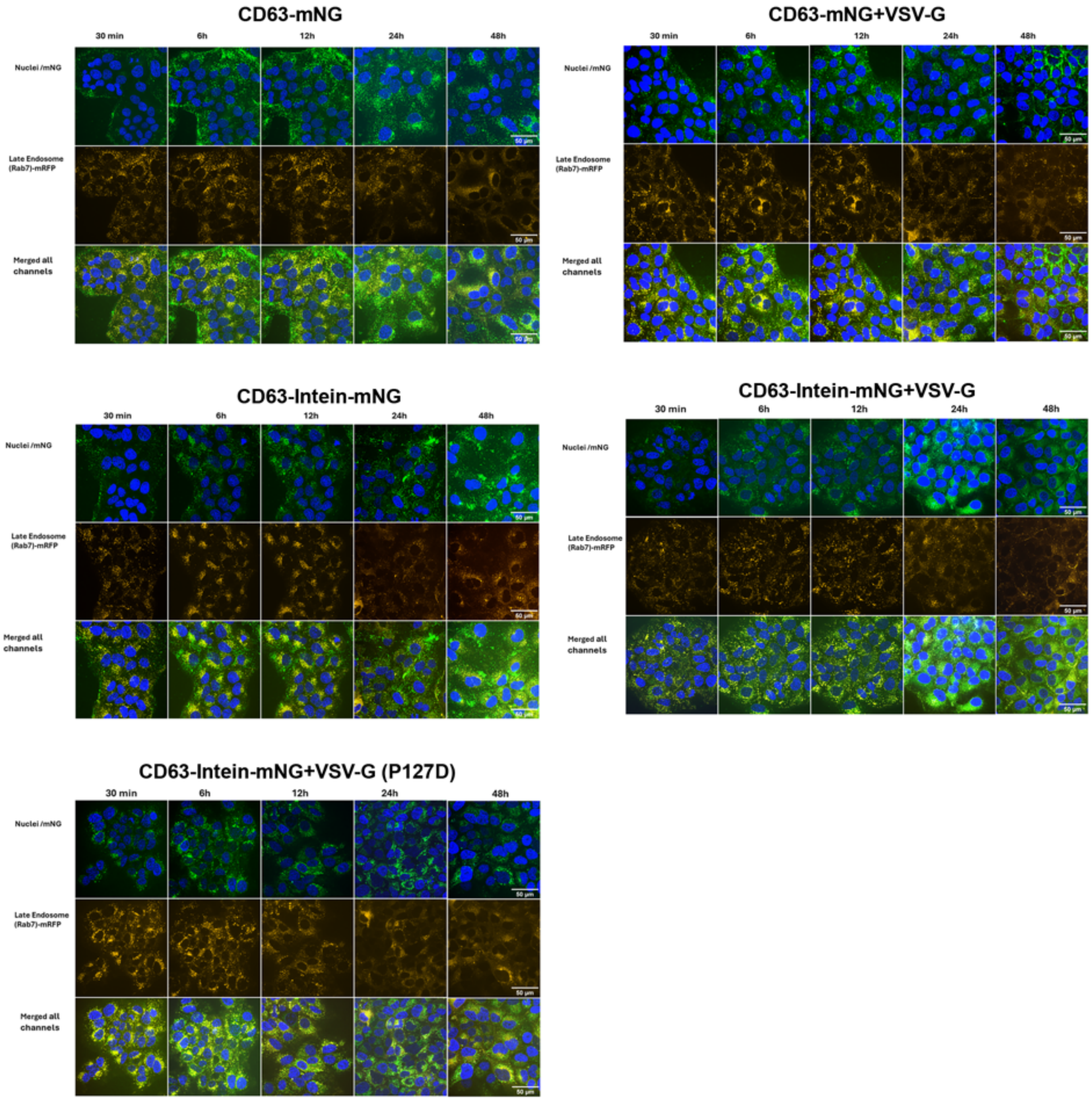

Supplementary Fig. 8. Co-localization of late endosome marker (Rab7) with the mNG in Huh7 cells at different time points (dynamic change) after adding engineered EVs. Rab7 was co-localized with mNG in different engineering groups at indicated time points.

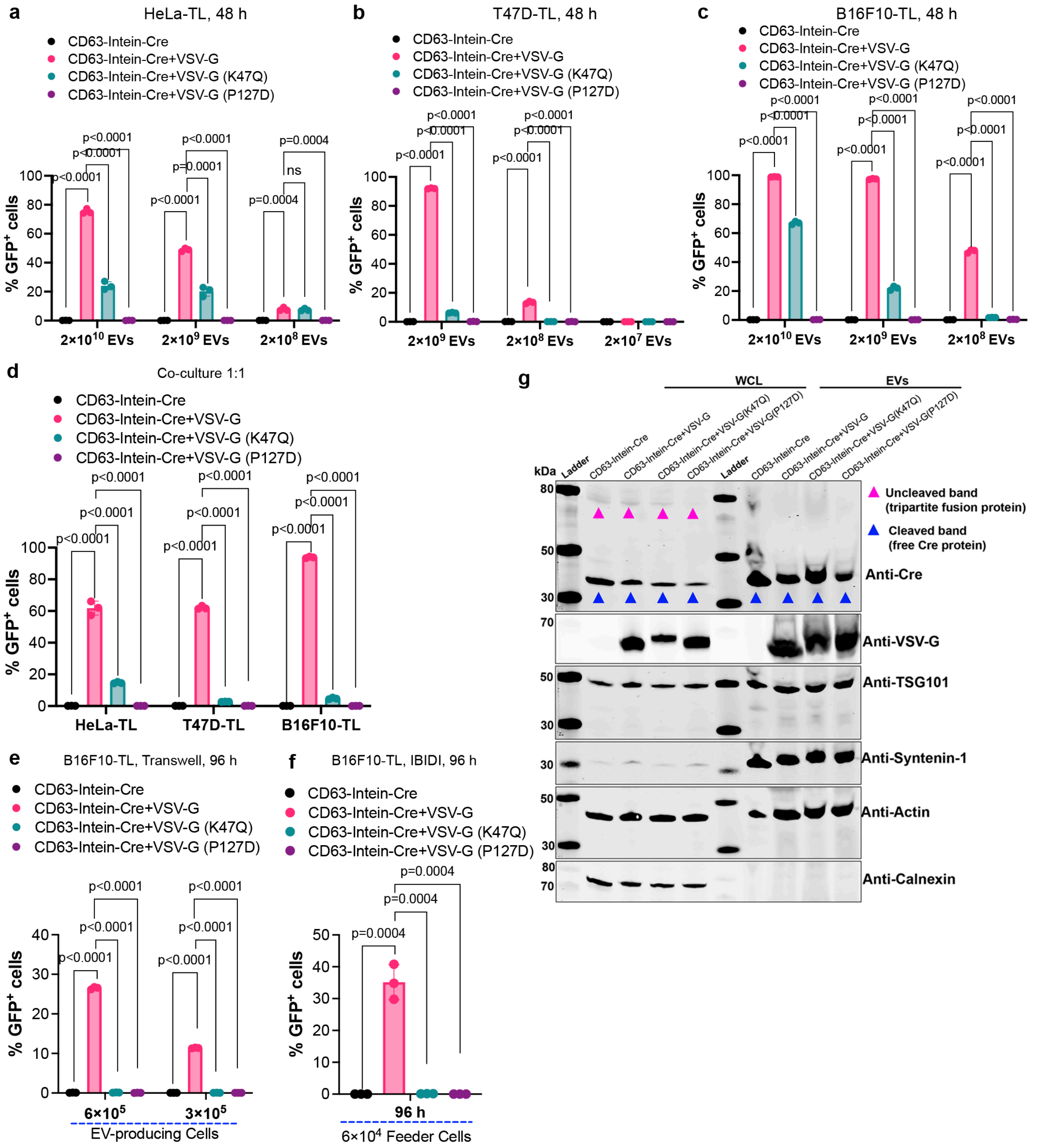

Supplementary Fig. 9. VSV-G boosts endosomal escape following receptor-mediated endocytosis of engineered EVs into recipient cells. a-c, Percentage of GFP positive HeLa-TL, T47D-TL and B16F10-TL cells after adding CD63-Intein-Cre EVs or wild type, P127D or K47Q VSV-G plus CD63-Intein-Cre EVs, as evaluated by flow cytometry. **d,** Direct co-culture assay to show the recombination efficiency of different engineered EVs. VSV-G (K47Q) and VSV-G (P127D) mutants were expressed in the EV-producing cells and resulting in abolished Cre delivery. **e,** Percentage of recombined GFP positive cells after a Transwell assay with B16F10-TL cells. VSV-G (K47Q) and VSV-G (P127D) mutants were expressed in the EV producing cells. Indicated numbers of EV-producing cells and 5×10^4 B16F10-TL cells were used for the assay in 24-well plates. **f,** Percentage of GFP positive cells evaluated by IBIDI assay in B16F10-TL cells. Indicated number of EV-producing cells and 4×10^4 reporter cells were used for this assay. **g,** Protein expression of mutated VSV-G-related constructs both in WCL of and isolated EVs evaluated by WB analysis. Proteins from 5×10^5 EV producing cells and 1×10^{10} engineered vesicles were used for the assay. TSG101, syntenin-1 and β-actin

were used as EV markers and Calnexin was used as cellular organelle marker (endoplasmic reticulum). Two-way ANOVA (Tukey) multiple comparisons test was used for analysis of (**a-f**). Experiments were done with 3 biological replicates and data are shown as mean±SD. Exact p values (p < 0.0001) were reported in the Source Data. Source data are provided as a Source Data file.

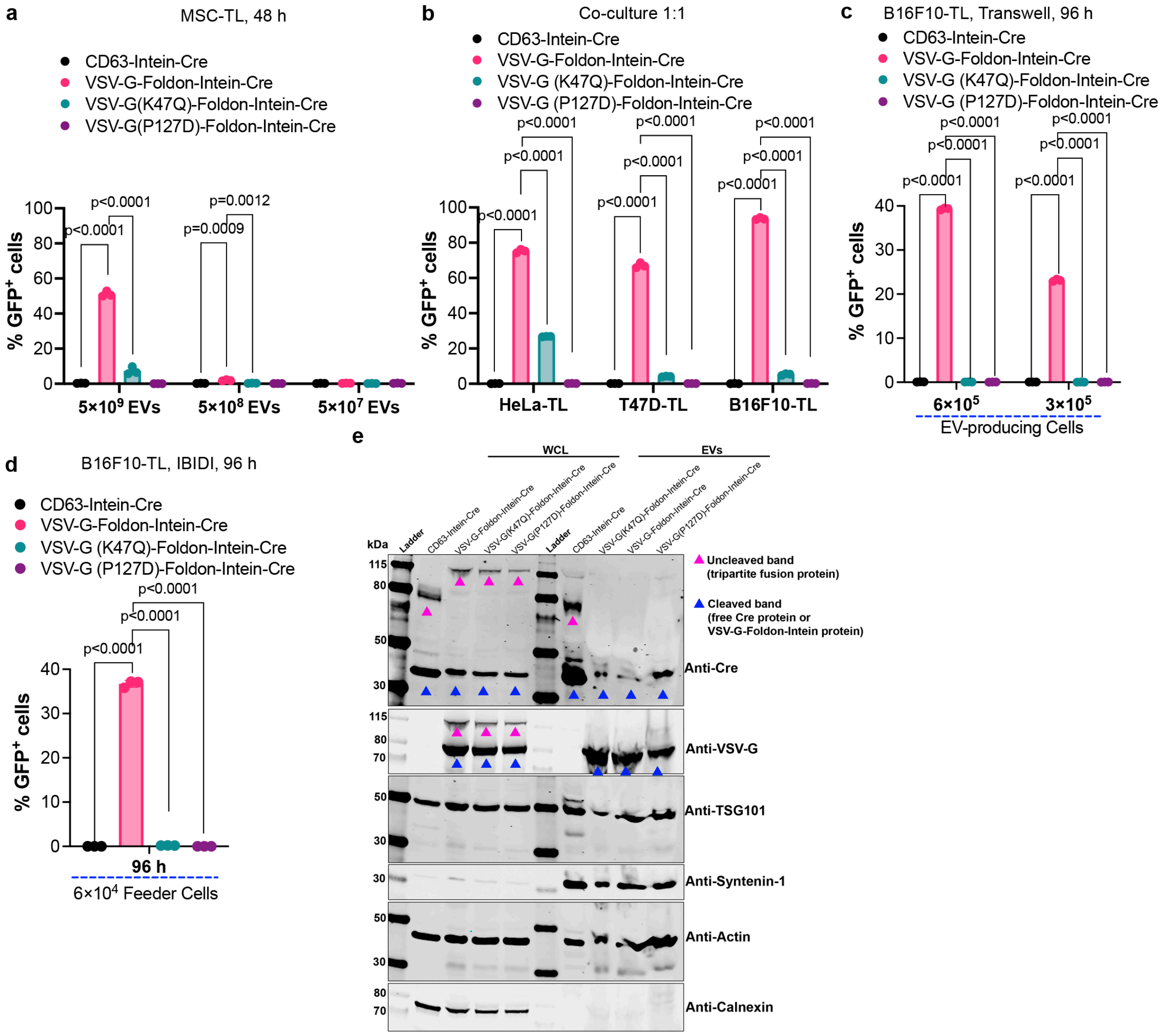

Supplementary Fig. 10. VSV-G boosts endosomal escape following receptor-mediated endocytosis of engineered EVs into recipient cells. a, Percentage of GFP positive MSC-TL cells after adding wild type, P127D or K47Q VSV-G directly fused with Foldon-Intein-Cre EVs, as evaluated by flow cytometry. **b,** Direct co-culture assay to show the recombination efficiency of different engineered EVs. VSV-G (K47Q) and VSV-G (P127D) mutants were expressed in the EV-producing cells and resulting in abolished Cre delivery. **c,** Percentage of recombined GFP positive cells after a Transwell assay with B16F10-TL cells. VSV-G (K47Q) and VSV-G (P127D) mutants were expressed in the EV producing cells. Indicated numbers of EV-producing cells and 5×10^4 B16F10-TL cells were used for the assay in 24-well plates. **d,** Percentage of GFP positive cells evaluated by IBIDI assay in B16F10-TL cells. Indicated number of EV-producing cells and 4×10^4 reporter cells were used for this assay. **e,** Protein expression of directly fused mutated VSV-G-related constructs both in WCL of and isolated EVs evaluated by WB analysis. Proteins from 5×10^5 EV producing cells and 1×10^{10} engineered vesicles were used for the assay. TSG101, syntenin-1 and β-actin were used as EV markers and Calnexin was used as cellular organelle marker (endoplasmic reticulum). Two-way

ANOVA (Tukey) multiple comparisons test was used for analysis of (**a-d**). Experiments were done with 3 biological replicates and data are shown as mean±SD. Exact p values ($p < 0.0001$) were reported in the Source Data. Source data are provided as a Source Data file.

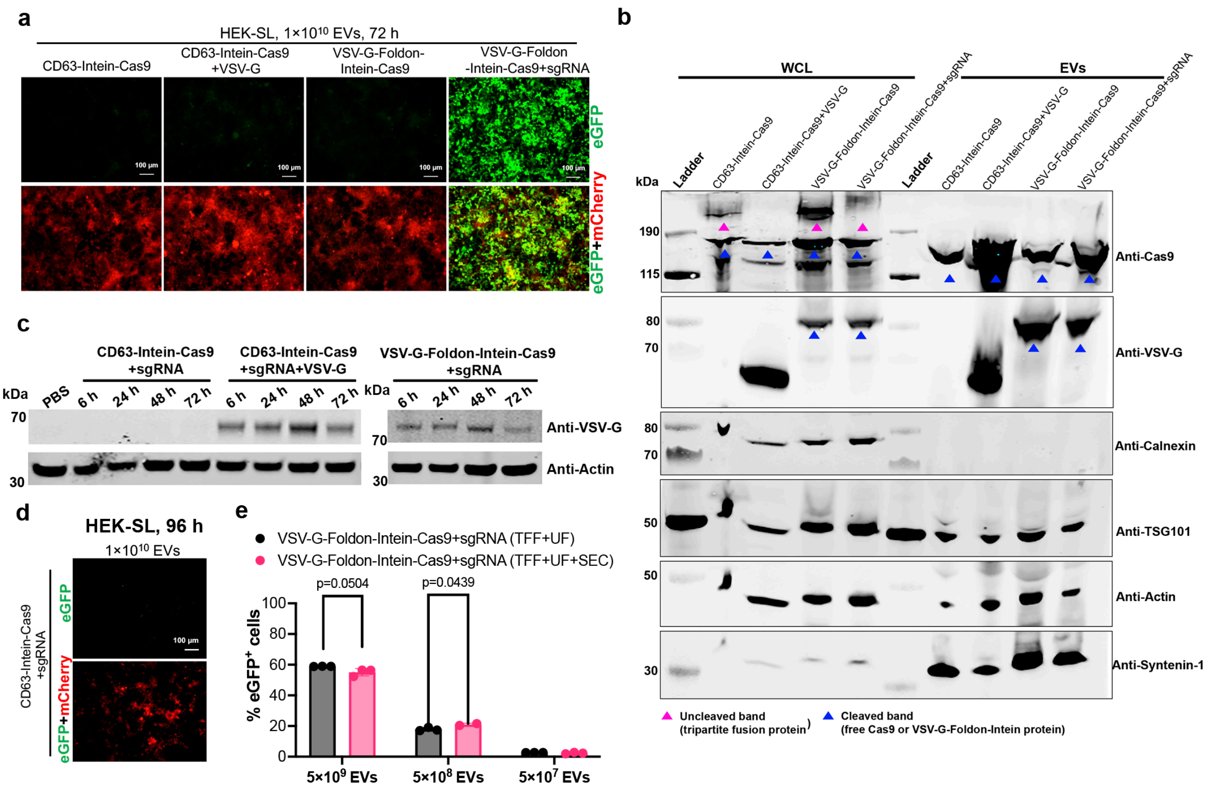

Supplementary Fig. 11. Characterization of Cas9-mediated genome editing in reporter cells by engineered EVs. a, Fluorescence microscopy images demonstrating functional Cas9/sgRNA RNPs delivery, as indicated by GFP positive signals, in HEK293T stoplight (HEK-SL) reporter cells after addition of indicated doses of EVs for 72 h. Scale bar, 100 µm, representative images. **b,** Protein expression of Cas9 related constructs in both whole cell lysate (WCL) and EVs evaluated by western blot analysis. Proteins from 5×10^5 EV-producing cells and 1×10^{10} engineered vesicles were used for the assay. TSG101, syntenin-1 and β-actin were used as EV markers while Calnexin was used as cellular organelle marker (endoplasmic reticulum). **c,** Dynamic changes of VSV-G protein from different EV groups in HEK-SL cells determined by western blot analysis. **d,** eGFP positive HEK-SL cells after adding CD63-Intein-Cas9+sgRNA EVs (without VSV-G as the negative control). Representative images, scale bar, 100 µm. **e,** Direct comparison of the editing efficiency of VFIC EVs in HEK-SL cells (96 h) isolated by TFF+UF and TFF+UF+SEC respectively. Two-way ANOVA (Tukey) multiple comparisons test was used for analysis of (**e**). Experiments were done with 3 biological replicates except the VSV-G-Foldon-Intein-Cas9+sgRNA (TFF+UF+SEC) 5×10^8 EV group which had 2 biological replicates for (**e**) and data are shown as mean±SD. Exact statistical analysis was reported in the Source Data. Source data are provided as a Source Data file.

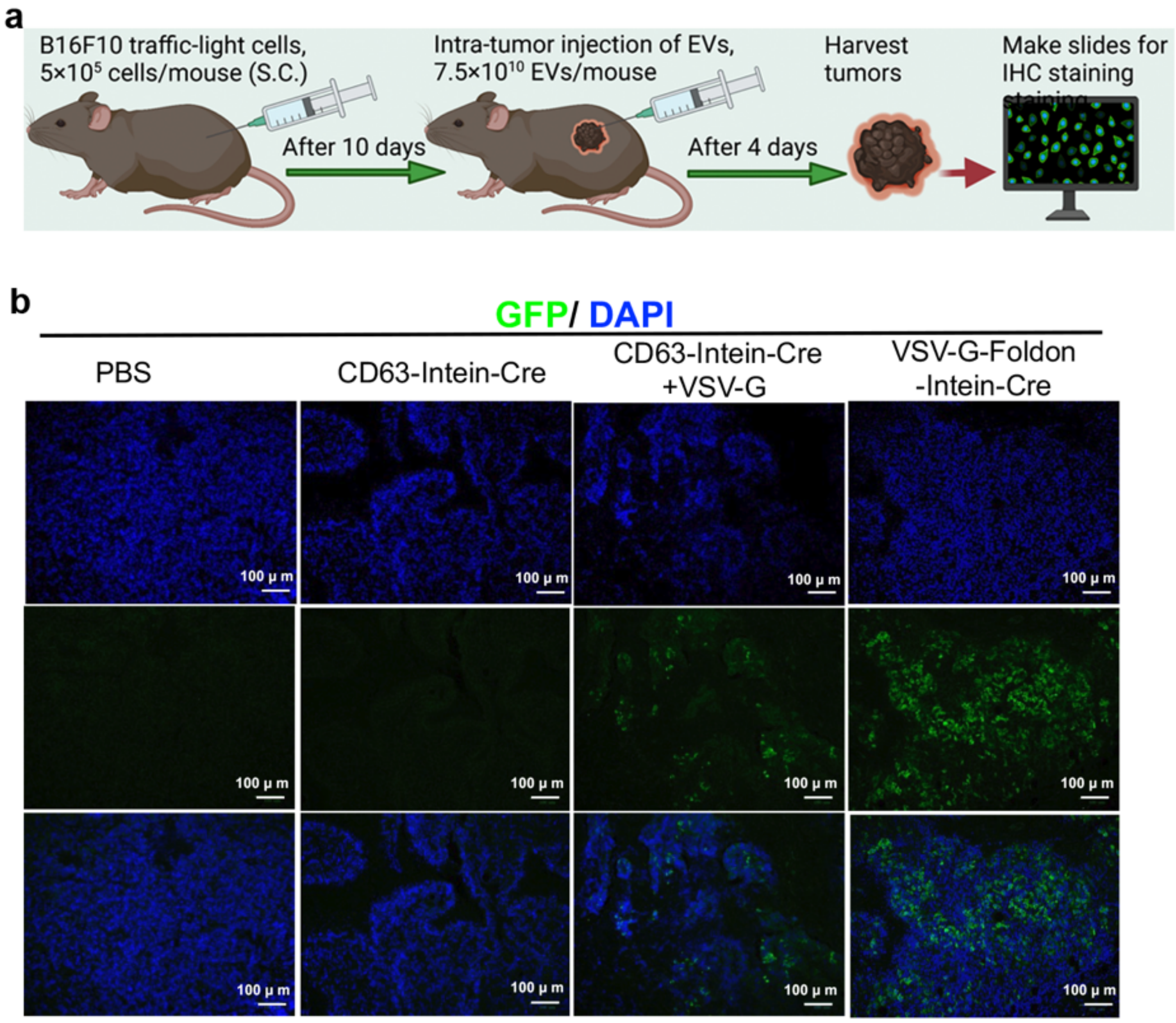

Supplementary Fig. 12. Cre recombination in melanoma-xenograft. a, Workflow for the intratumoral injection model. **b,** Representative immunofluorescence images from tumor tissues after intratumoral injection of engineered EVs to induce GFP expression. Scale bar, 100 µm. n=3 mice per group. **a** Created in BioRender.com, Zheng, W. (2025) *https://BioRender.com/o78k534*.

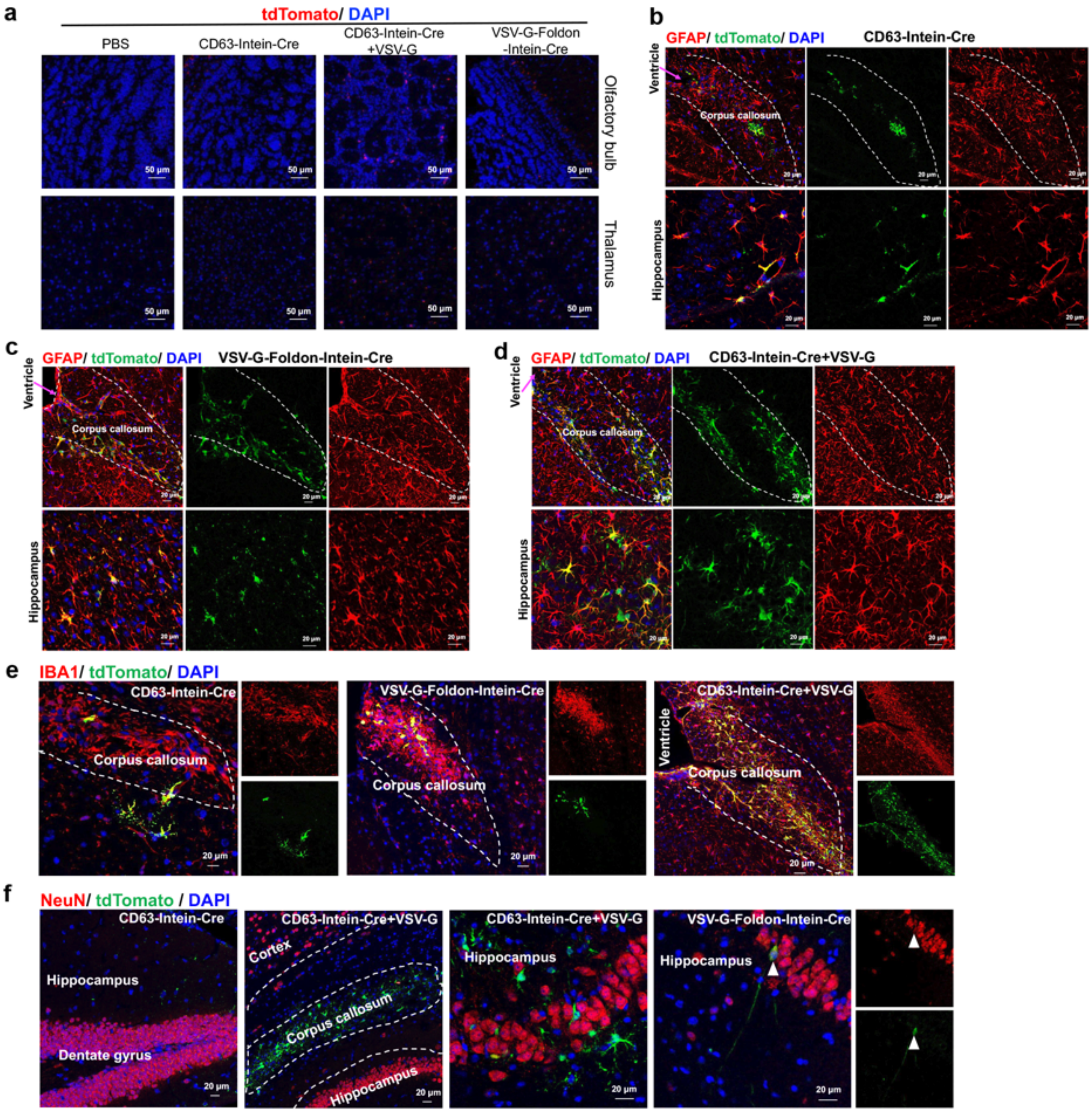

Supplementary Fig. 13. Cre-mediated recombination in R26-LSL-tdTomato reporter mice by VEDIC and VFIC systems after local injection. a, IHC staining of the olfactory bulb and thalamus one week after ICV injection of the engineered EVs. Scale bar, 50μm. **b-d,** Co-staining of tdTomato with the astrocyte marker GFAP in corpus callosum (outlined by dashed lines) and hippocampus one week after ICV injection of engineered EVs. Scale bar, 20 μm. **e,** Co-staining of microglia marker IBA1 with tdTomato in corpus callosum (highlighted by dashed lines) after ICV injection of engineered EVs. Scale bar, 20 μm. **f,** Co-staining of neuron cell marker NeuN with tdTomato in different regions of brain after ICV injection of different engineered EVs. Scale bar, 20 μm. White arrow indicates sporadic cells with co-localization of NeuN and RFP. n=3 mice for PBS and n=4 for other groups, representative images for (**a**-**f**).

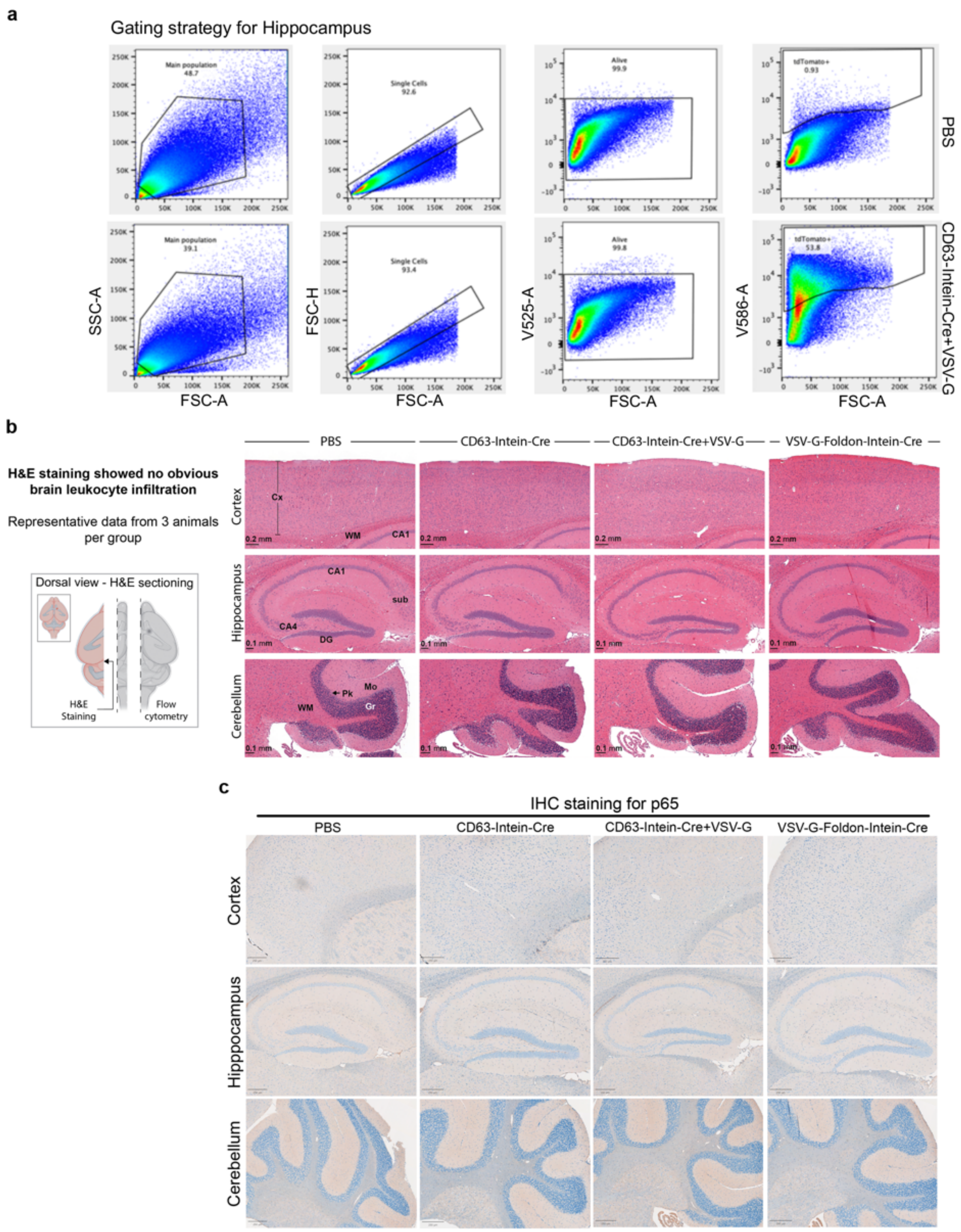

Supplementary Fig. 14. Gating strategy of FACS for evaluating tdTomato positive cells, H&E and IHC staining of p65 from different regions of brain after minipump ICV injections. a, Representative gating strategy of FACS to measure the tdTomato positive cells from different regions of brain after minipump ICV injections. **b,** H&E staining to show the infiltration of inflammatory cells in different regions of brain after minipump ICV injections. Scale bar, 200 µm for cortex and 100 µm for hippocampus and cerebellum, representative images. Hematoxylin-Eosin (H&E) for brain sagittal sections. (Cx) cortex; (WM) white matter; Ammon´s horn divided in CA1- CA4 regions; (DG) dentate gyrus; (sub) subiculum; (Mo) molecular layer; (Pk) Purkinje cell layer and (Gr) granular layer. **c,** IHC staining of p65 to demonstrate

activation of inflammation in different regions of brain after minipump ICV injections. Scale bar, 250 μm, representative images.

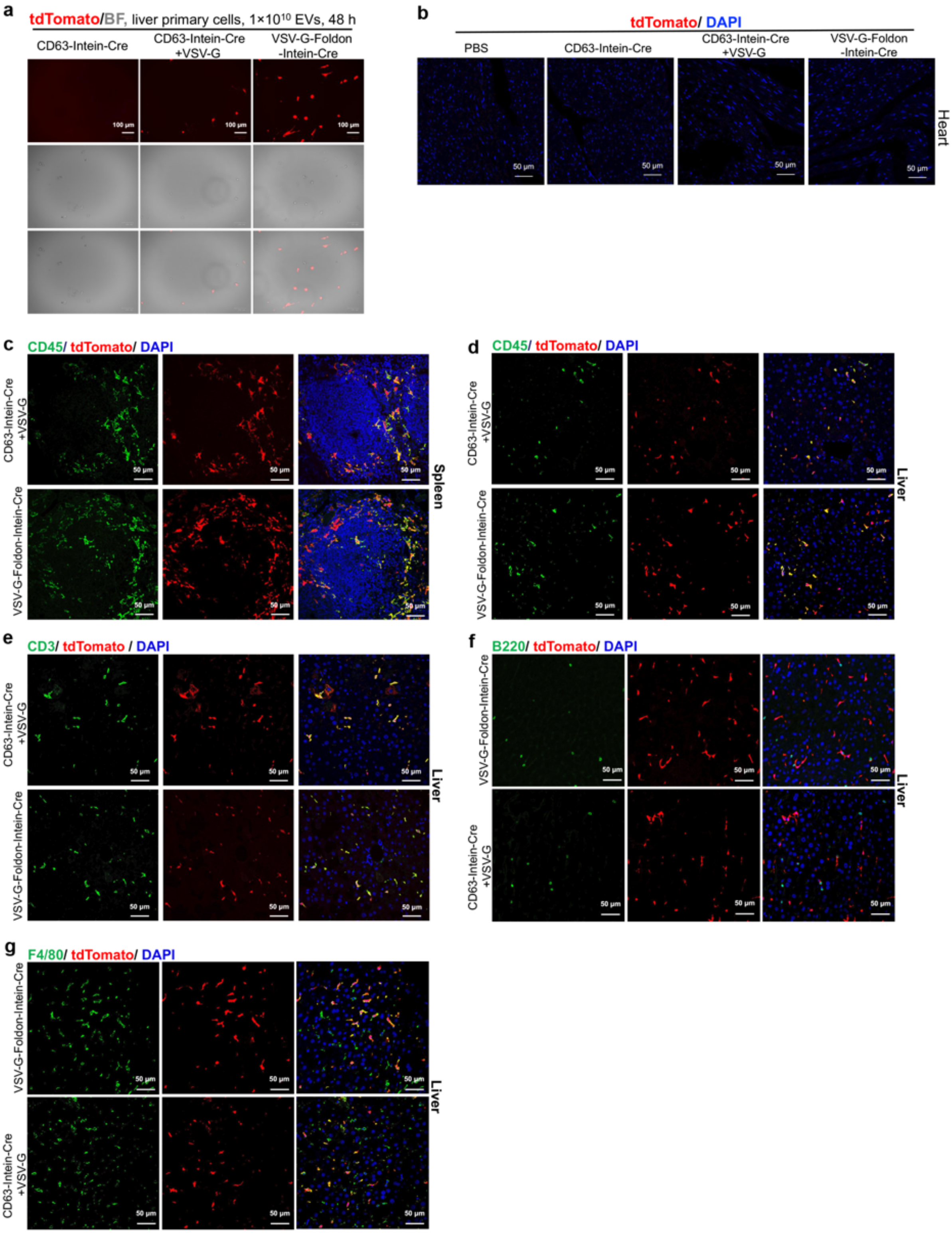

Supplementary Fig. 15. Cre recombination in R26-LSL-tdTomato reporter mice following systemic VEDIC and VFIC EV-mediated Cre delivery. a, Representative fluorescent microscopic images demonstrated tdTomato positive liver primary cells from R26-LSL-tdTomato reporter mice after adding VEDIC and VFIC EVs for 2 days. Scale bar, 100 μm. **b,** IHC staining of the heart, one week after IP injection of the engineered EVs. Scale bar,

50 µm. **c,** Co-staining of tdTomato with the general leukocyte marker CD45 in spleen as detected by immunofluorescence one week after IP injection of engineered EVs. Scale bar, 50 µm. **d,** Co-staining of general leukocyte marker CD45 with tdTomato expression in liver after IP injection of engineered EVs. Scale bar, 50 µm. **e,** C-localization of T cell marker CD3 with tdTomato in liver one week after IP injection of engineered EVs as detected by IHC co-staining. Scale bar, 50 µm. **f,** Co-localization of B cell marker B220 with tdTomato in liver one week after IP injection of different engineered EVs. Scale bar, 50 µm. **g,** Co-expression of macrophage marker F4/80 with tdTomato in liver evaluated by IHC co-staining one week after injecting engineered EVs. Scale bar, 50 µm. n=3 mice per group, representative images for (**b-g**).

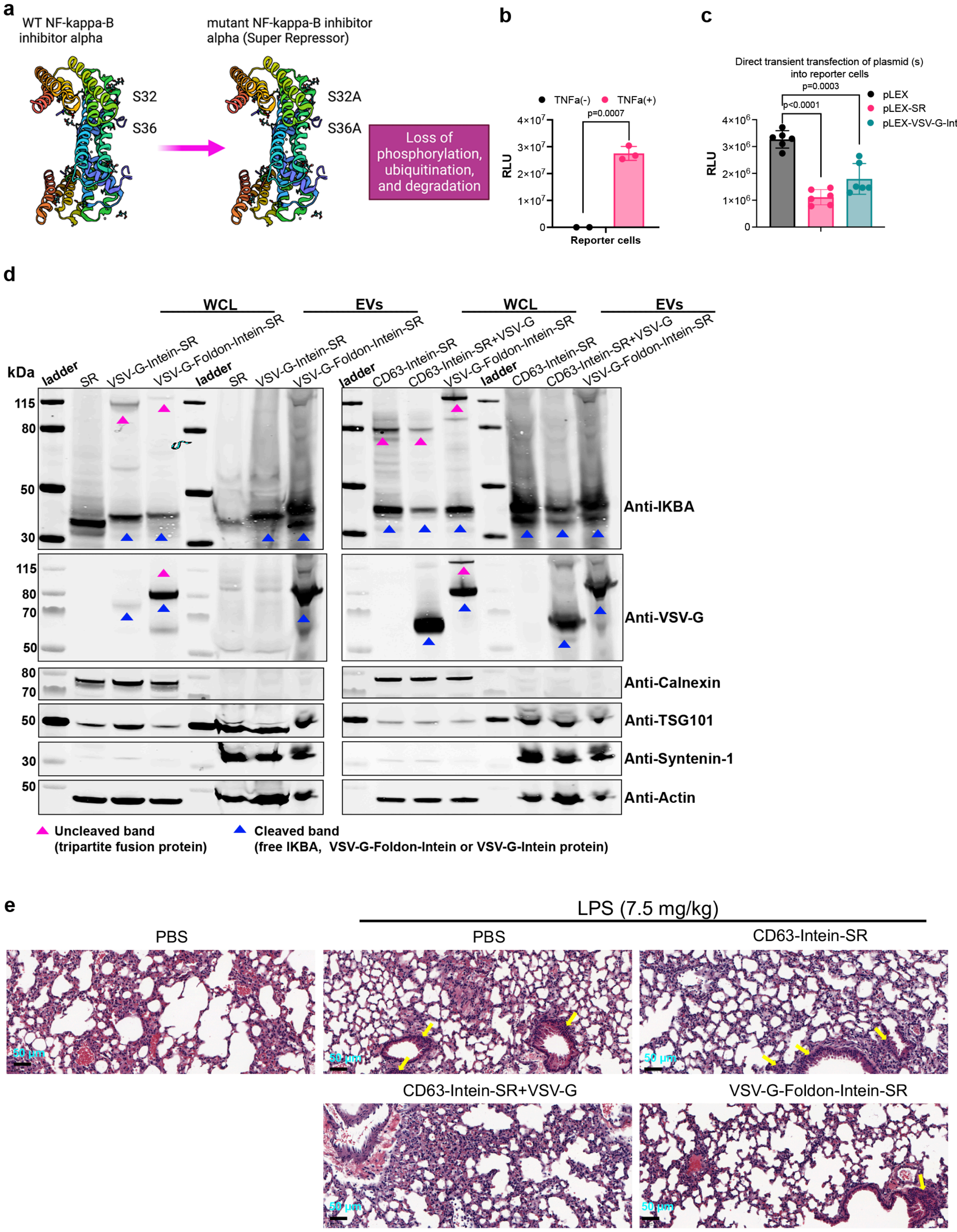

Supplementary Fig. 16. The design of super repressor (SR) inhibitor of NF-κB constructs and their expression and function in vitro. a, The mutations and properties of the super repressor inhibitor of NF-κB. **b,** Activation of luciferase expression 6 h after TNF-α stimulation (10 ng/ml) in HEK-Blue-NF-κB reporter cells. **c,** Significant decrease of the luciferase signals from lysate of HEK-Blue-NF-κB reporter cells after transient transfection of SR-related constructs. 48 h after transfection cells were stimulated with TNF-α for 6 h prior to luciferase detection. **d,** Protein expression of SR-related constructs in both WCL and isolated EVs evaluated by WB analysis. Proteins from 5×10^5 EV-producing cells and 1×10^{10} engineered vesicles were used for the assay. TSG101, syntenin-1 and β-actin were used as EV markers while Calnexin was used as cellular organelle marker (endoplasmic reticulum). **e,** Infiltration of inflammatory cells in lung tissues as shown by the representative histology images (yellow arrows indicate the inflammatory cells). Scale bar, 50 µm. Two-tailed T-test was used for the analysis of (**b**); One-way ANOVA (Tukey) multiple comparisons test was used for analysis of (**c**). **a** Created in BioRender.com, Zheng, W. (2025) *https://BioRender.com/z26t488*. Experiments were done with 2 biological replicates TNF-α (-) of (**b**), 3 biological replicates TNF-α (+) of (**b**), and 6 biological replicates for (**c**), and data are shown as mean±SD. Exact p values ($p < 0.0001$) were reported in the Source Data. Source data are provided as a Source Data file.

Supplementary Table 1: primary and secondary antibodies for IHC.

Reagent or resource	Source	Identifier	Dilution
Primary antibodies for IHC			
Rabbit anti-IBA1	Wako	Cat#: 019-1941	1:500
Rabbit anti-GFAP	Abcam	Cat#: ab53554	1:500
Rabbit anti-RFP	Thermo Fisher Scientific	Cat#: 600-401-379-RTU	1:400
Biotin anti-RFP	Abcam	Cat#: ab34771	1:400
Rabbit anti-CD45	Abcam	Cat#: ab10558	1:200
Rat anti-F4/80	Bio-Rad	Cat#: MCA497G	1:100
Rat anti-B220	Thermo Fisher Scientific	Cat#: 14-0452-82	1:100
Rat anti-NeuN	Millipore	Cat#: MAB377	1:100
Rat anti-CD3	Dako	Cat#: A0452	1:100
Rat anti-Mouse CD8a	BD Pharmingen	Cat#: 553027	1:500
Rabbit anti-GFP	Abcam	Cat#: ab290	1:200
Rabbit anti-Mouse NF-kB p65	Abcam	Cat#: ab16502	1:4000
Secondary antibodies for IHC			
Alexa Flour 488 goat anti-mouse	Thermo Fisher Scientific	Cat#: A11001	1:400
Alexa Fluor 633 goat anti-rabbit	Thermo Fisher Scientific	Cat#: A21070	1:400
Alexa Fluor 568 goat anti-rat	Thermo Fisher Scientific	Cat#: A11077	1:400
Alexa Fluor 568-conjugated streptavidin	Thermo Fisher Scientific	Cat#: S11226	1:400
Alexa Fluor 488 goat anti-rat	Abcam	Cat#: ab150077	1:800

Supplementary Table 2: primary and secondary antibodies for WB.

Reagent or resource	Source	Identifier	Dilution
Primary antibodies for WB			
Rabbit anti-TSG101	Thermo Fisher Scientific	Cat#: PA5-82236	1:1000
Rabbit anti-Cre	Abcam	Cat#: ab188568	1:1000
Rabbit anti-Syntenin-1	Thermo Fisher Scientific	Cat#: 600-401-379-RTU	1:1000
Goat anti-VSV-G	Thermo Fisher Scientific	Cat#: PA1-30278	1:1000
Mouse anti-Cas9	Thermo Fisher Scientific	Cat#: MA5-23519	1:1000
Goat anti-Calnexin	Thermo Fisher Scientific	Cat#: MCA497G	1:1000
Mouse anti-β-Actin	Sigma	Cat#: A5441	1:20000
Mouse anti- IkB-alpha	Thermo Fisher Scientific	Cat#: MA5-15132	1:1000
Rabbit anti-PCSK9	Thermo Fisher Scientific	Cat#: PA5-78663	1:1000
Secondary antibodies for WB			
IRDye® 800CW Donkey anti-Goat IgG	LI-COR Biosciences	Cat#: 926-32214	1:10000
IRDye® 800CW Goat anti-Mouse IgG	LI-COR Biosciences	Cat#: 926-32210	1:10000
IRDye® 800CW Goat anti-Rabbit IgG	LI-COR Biosciences	Cat#: 926-32211	1:10000
IRDye® 680RD Goat anti-Mouse IgG	LI-COR Biosciences	Cat#: 926-68070	1:10000
IRDye® 800CW Donkey anti-Goat IgG	LI-COR Biosciences	Cat#: 926-32214	1:10000

Supplementary Table 3: plasmids used in this study (1).

Reagent or resource (plasmids)	Source	Identifier
pCMV-VSV-G	Addgene	8454
pLV-CMV-LoxP-DsRed-LoxP-eGFP	Addgene	65726
pCMV-VSV-G-(P127D)-Myc	Addgene	80055
pBIC-Gag-CAS9	Addgene	119942
pBS-CMV-gagpol	Addgene	35614
pLEX-Intein-Cre	This study	
pLEX-CD63-Cre	This study	
pLEX-CD63-Intein-Cre	This study	
pLEX-VSV-G-Intein-Cre	This study	
pLEX-VSV-G-Cre	This study	
pLEX-VSV-G-Foldon-Intein-Cre	This study	
pLEX-VSV-G-Foldon-Intein-MS2	This study	
pLEX-VSV-G-Foldon-Cre	This study	
pLEX-CD63-mnGFP	This study	
pLEX-CD63-Intein-mnGFP	This study	
pLEX-VSV-G (K47Q)-Foldon-Intein-Cre	This study	
pLEX-VSV-G (P127D)-Fodon-Intein-Cre	This study	
pLEX-VSV-G (K47Q)	This study	
pLEX-VSV-G-Foldon-Intein-Cas9	This study	
pLEX-CD63-Intein-Cas9	This study	
pLEX-VSV-G-Intein-SR	This study	
pLEX-VSV-G-Foldon-Intein-SR	This study	
pLEX-super repressor (SR)	This study	
pLEX-CD9-Intein-Cre	This study	
pLEX-C81-Intein-Cre	This study	
pLEX-PTGFRN-Intein-Cre	This study	
pBB-U6-GFPsgRNA	This study	
pLEX-CV-G-Intein-Cre	This study	
pLEX-EPFV-Intein-Cre	This study	
pLEX-CV-G	This study	
pTwist CMV-ERVK-19	This study	
pTwist CMV-ERVMER34-1	This study	
pTwist CMV-ERVI-1	This study	
pTwist CMV-SPACA3	This study	
pTwist CMV-EQTN	This study	
pTwist CMV-TMEM95	This study	
pTwist CMV-HERV-H	This study	
pTwist CMV-ERVV-2	This study	
pTwist CMV-ERVK13-1	This study	
pTwist CMV-FIMP	This study	
pTwist CMV-Syncytin-2	This study	
pTwist CMV-HERVK_113	This study	
pTwist CMV-ERVK-21	This study	
pTwist CMV-Env type 1	This study	
pTwist CMV-SPAM1	This study	
pTwist CMV-ERVK-18	This study	
pTwist CMV-ERVK-8	This study	
pTwist CMV-ERVK-25	This study	
pTwist CMV-ADAM12	This study	
pTwist CMV-ERVK-9	This study	
pTwist CMV-ERVK-24	This study	
pTwist CMV-Envelope glycoprotein (1)	This study	
pTwist CMV-IZUMO1	This study	
pTwist CMV-HERV-H	This study	

Supplementary Table 4: plasmids used in this study (2).

pTwist CMV-ERVK-7	
pTwist CMV-ERVS71-1	This study
pTwist CMV-Envelope protein	This study
pTwist CMV-Pol/env protein	This study
pTwist CMV-MYMK	This study
pTwist CMV-ERVK-6	This study
pTwist CMV-ERVPABLB-1	This study
pTwist CMV-Envelope polyprotein (2)	This study
pTwist CMV-FLJ32214	This study
pTwist CMV-SPACA6	This study
pTwist CMV-ERVFC1	This study
pTwist CMV-ERVV-1	This study
pTwist CMV-Envelope glycoprotein (3)	This study
pTwist CMV-Env type 2	This study
pTwist CMV-MYMX	This study
pTwist CMV-IZUMO1R	This study
pcoPE01(EPFV)	This study
pCD/NL-BH	This study
pLEX-M1 PCSK9	This study
pLEX-CD63-Intein-M1	This study
pLEX-VSV-G-Foldon-Intein-M1	This study
pLEX-CD63-Intein (H439Q)-Cre	This study
pLEX-CD63-Intein (N440A)-Cre	This study
pLEX-VSV-G-Foldon-Intein (H439Q)-Cre	This study
pLEX-VSV-G-Foldon-Intein (N440A)-Cre	This study
pLEX-CD63-Intein-SR	This study

Supplementary Table 5: sgRNA and primer sequences.

Reagent or resource (sequences)	Source	Sequence
sgRNA for HEK-SL	This study	GGACAGTACTCCGCTCGAGT
sgRNA for mTTR	This study	TTACAGCCACGTCTACAGCA
PCR primer for mTTR	This study	
Forward		gggatcagcatgtacttttggt
Reverse		cttacaagagcaatacgtgccc

Supplementary Table 6: Reagents and cell lines used in this study.

Reagent or resource	Source	Identifier
Plasmid Plus Midi Kit	QIAGEN	Cat#: 12943
FastScanTM Cas9 (S. pyogenes) ELISA Kit	Cell Signaling Technology	Cat#: 29666
Plasmid Plus Maxi Kit	QIAGEN	Cat#: 12963
Phusion High-Fidelity DNA Polymerase	Thermo Fisher Scientific	Cat#: 15331732
NucleoSpin Gel and PCR Clean-up XS	Techtum Lab	Cat#: 32-740611.250
HotStarTaq Plus Master Mix Kit	QIAGEN	Cat#: 203645
Maxwell RSC Cell DNA Purification Kit	Promega	Cat#: AS1370
T4 DNA Ligase	Thermo Fisher Scientific	Cat#: EL0011
One Shot TOP10 Chemically Competent E. coli	Thermo Fisher Scientific	Cat#: C404006
Ibidi USA U SLIDE	IBIDI	Cat#: 81806
Millicell Cell Culture Insert	Merck Millipore	Cat#: PICM01250
polyethylenimine (PEI) MAX	Polysciences	Cat#: 24765-1
InterceptTM blocking buffer	LI-COR Biosciences	Cat#: 927-60003
TNF-α	Thermo Fisher Scientific	Cat#: PHC3013

Reagent or resource	Source	Identifier
Experimental cell lines		
HEK-293T	ATCC	Cat#: CRL-3216
HeLa-TL	This study	
Huh7	Elabscience	Cat#: EP-CL-0120
B16F10-TL	This study	
MSC-TL	This study	
Raw264.7-TL	This study	
THP-1-TL	This study	
K562-TL	This study	
HEK-SL	Pieter Vader's lab	
MSC-TL	This study	
T47D-TL	This study	
HEK-NF-κB	InvivoGen	

Supplementary Table 7: Experimental animals used in this study.

Reagent or resource	Source	Identifier
Experimental animals		
C57BL/6J mice	Charles River Laboratories	Cat#: 027
Cre-LoxP R26-LSL-tdTomato reporter mice	Jackson Laboratory	Cat#: 007914

Unprocessed blots of Supplementary figures

Panels used in the paper

Fig. s1d: left panels without VSV-G

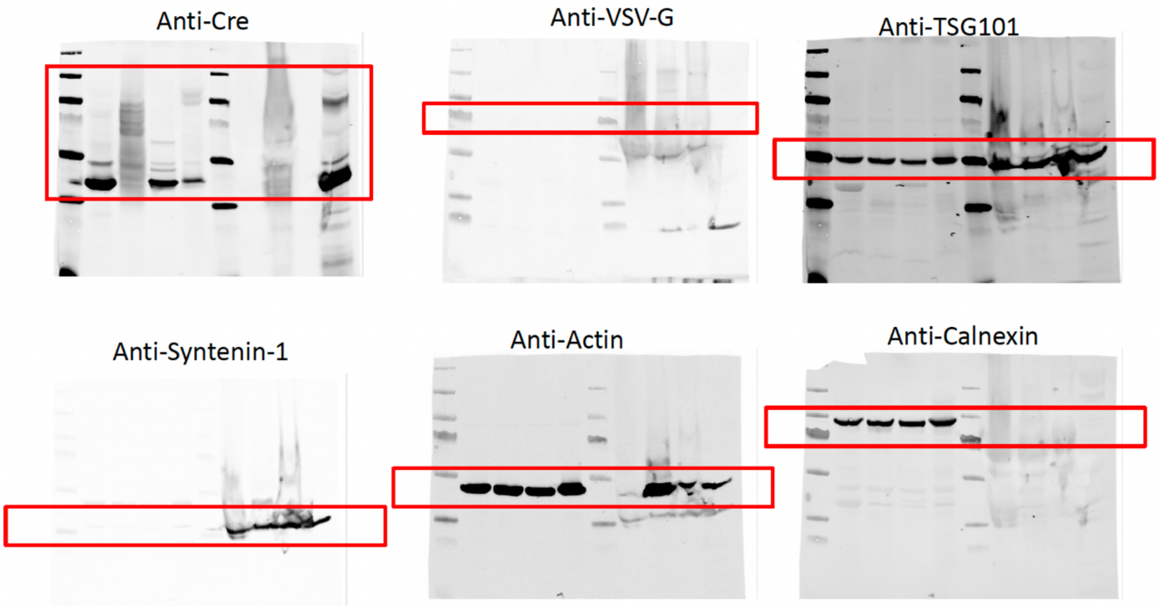

Fig. s1d: right panels with VSV-G

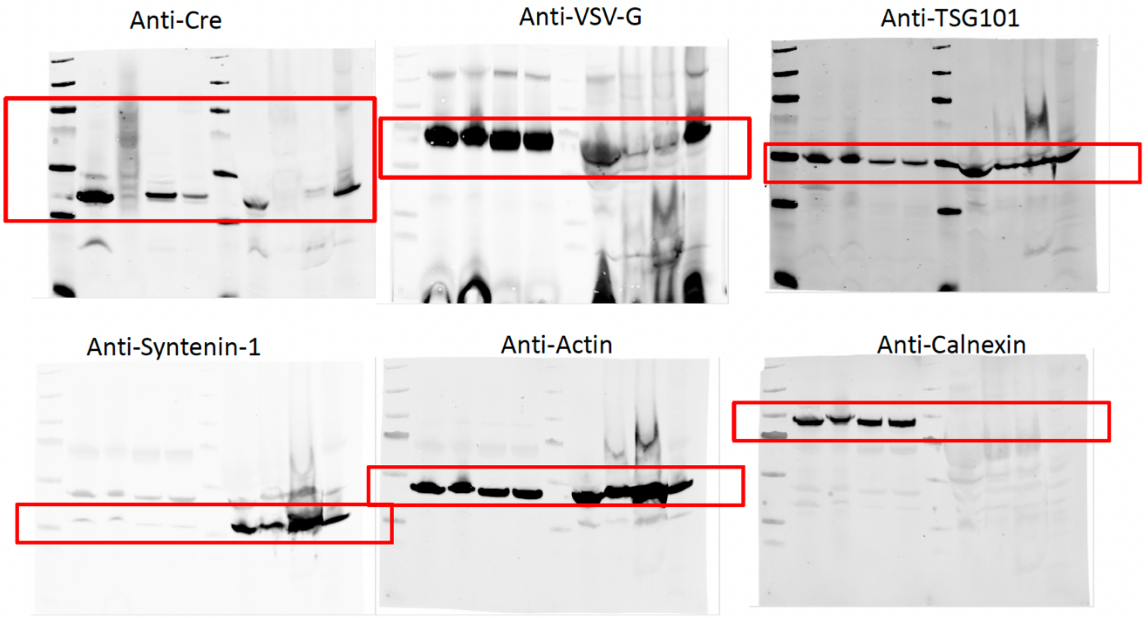

Fig. s2c

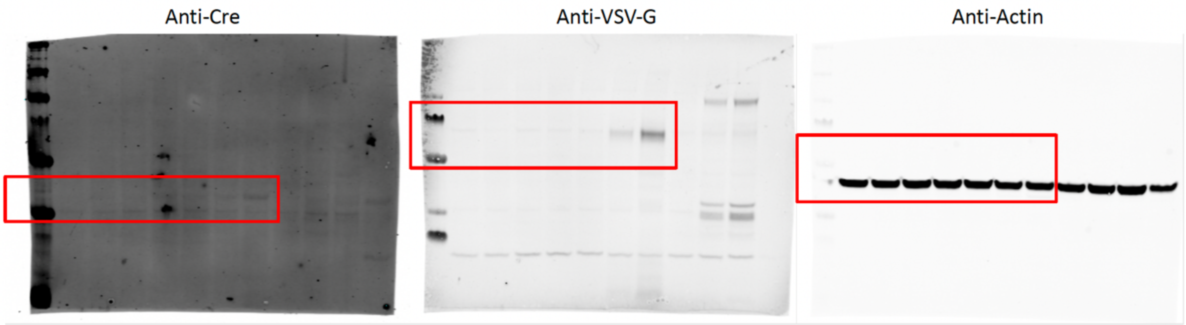

Fig. s4d: left panels

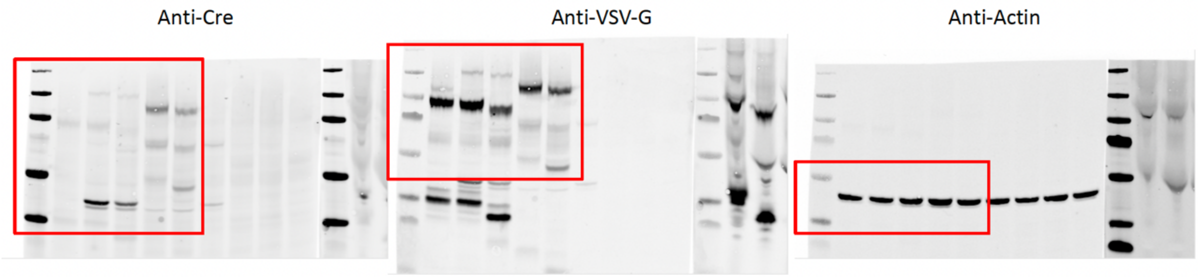

Fig. s4d: right panels

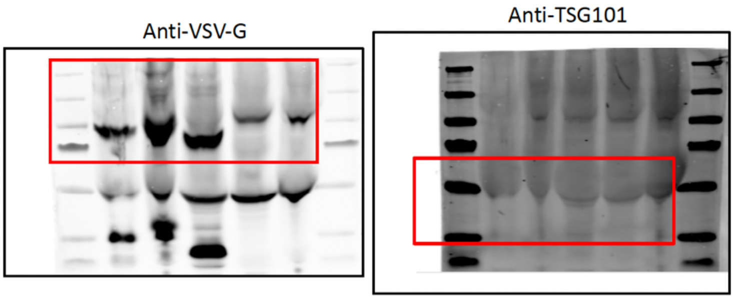

Fig. s6c

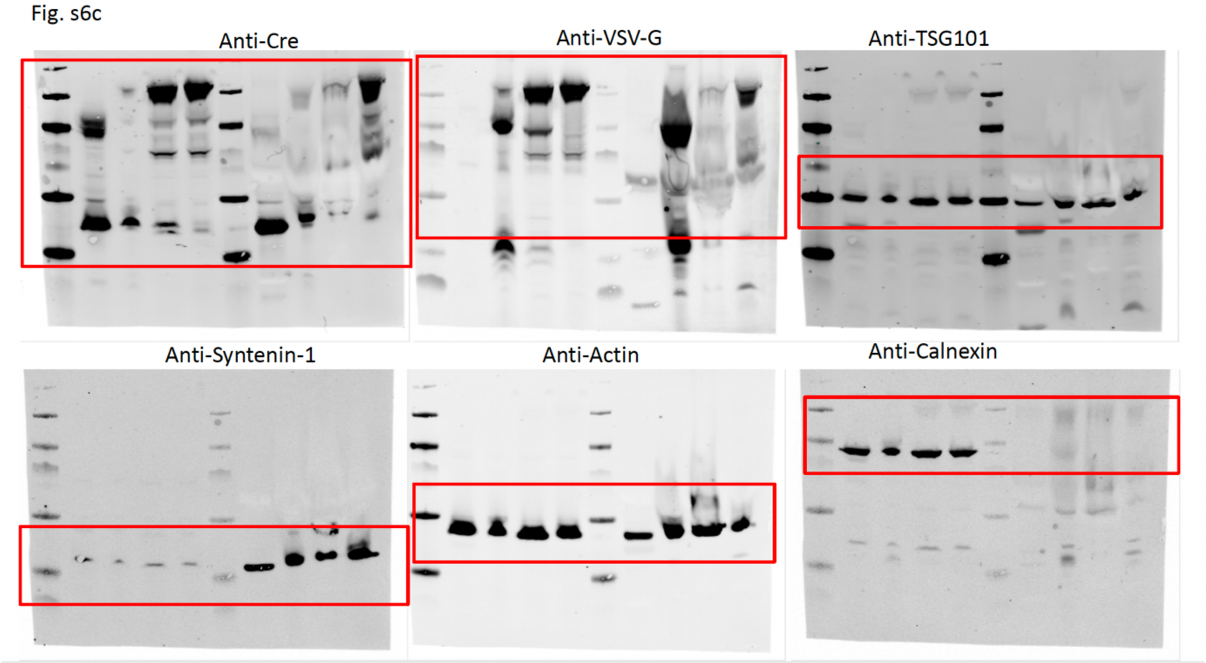

Fig. s9g

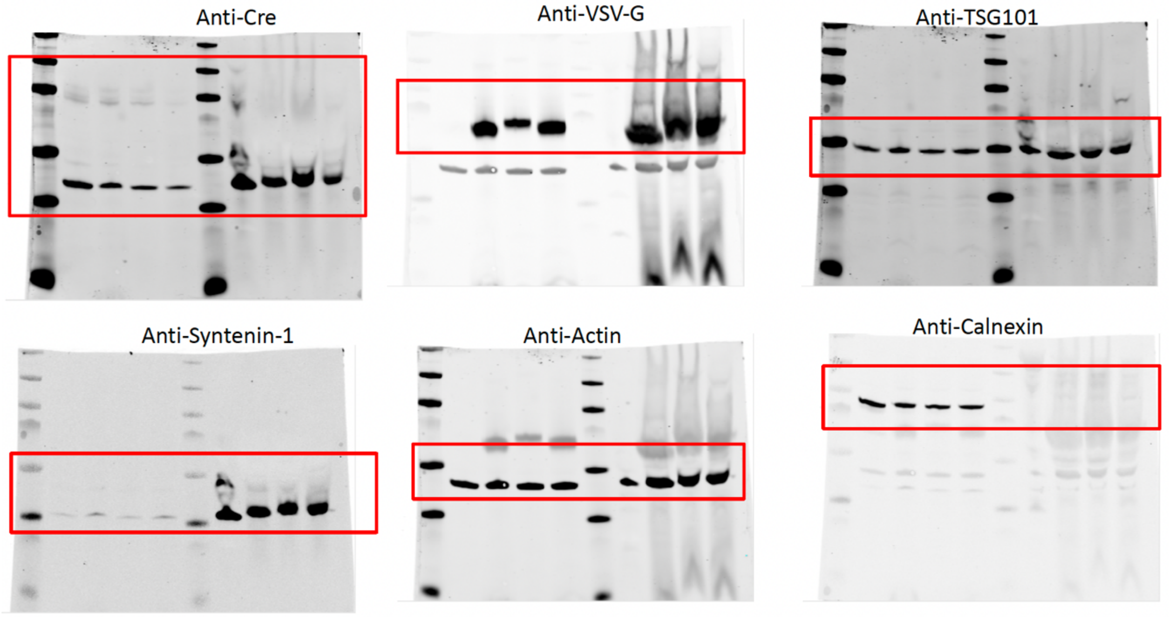

Fig. s10e

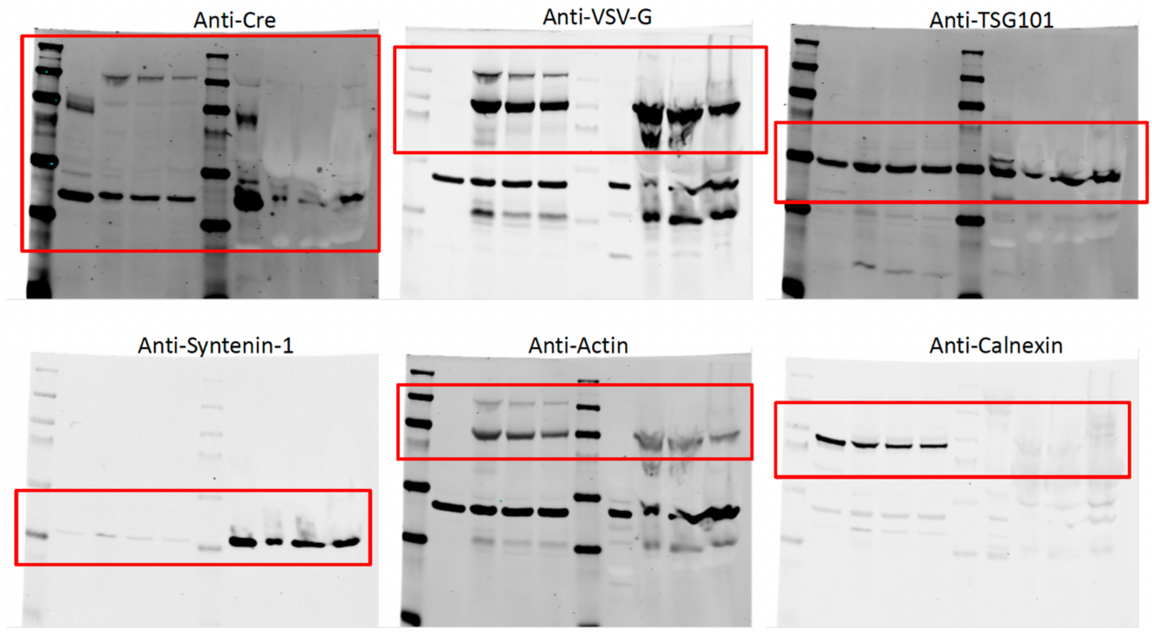

Fig. s11b

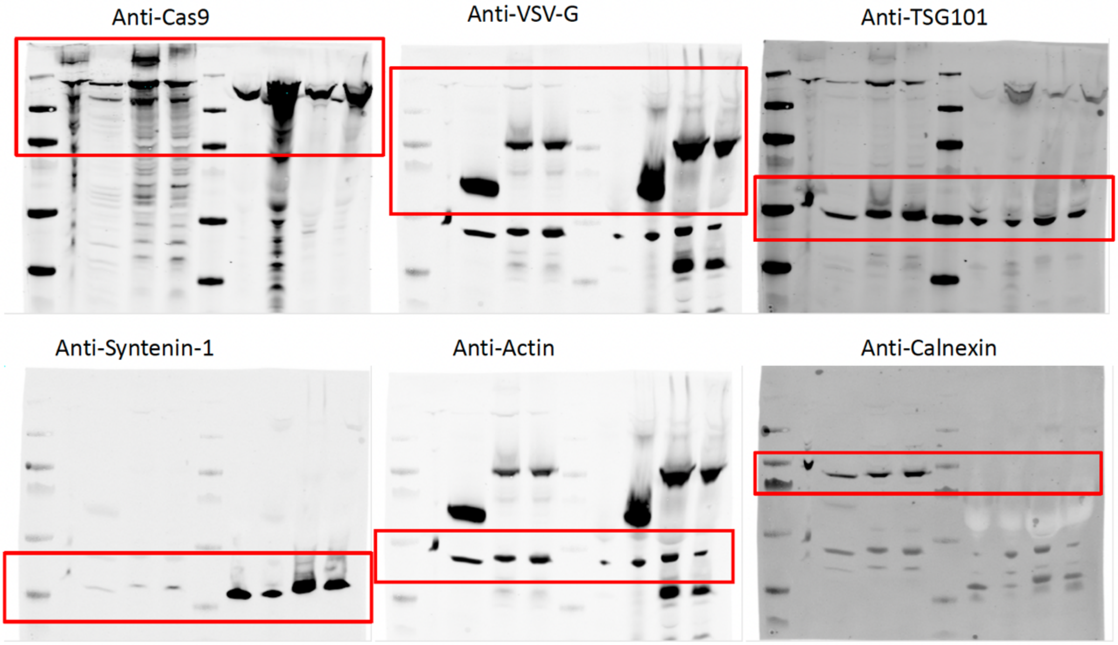

Fig. s11c

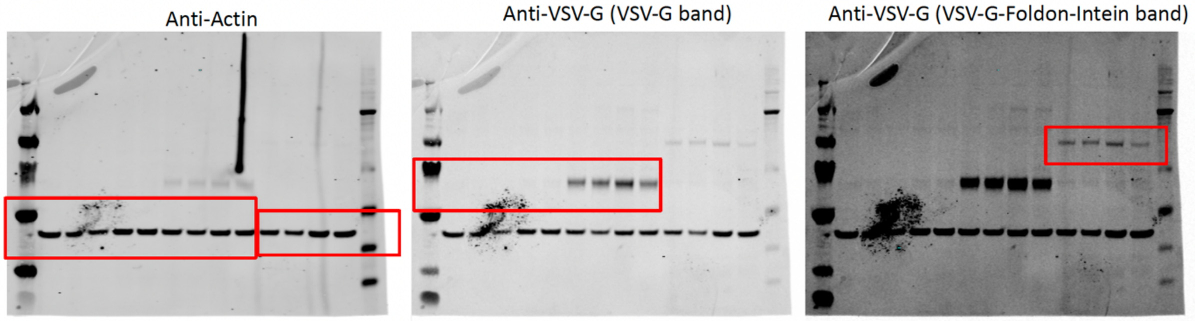

Fig. s16d: left panels

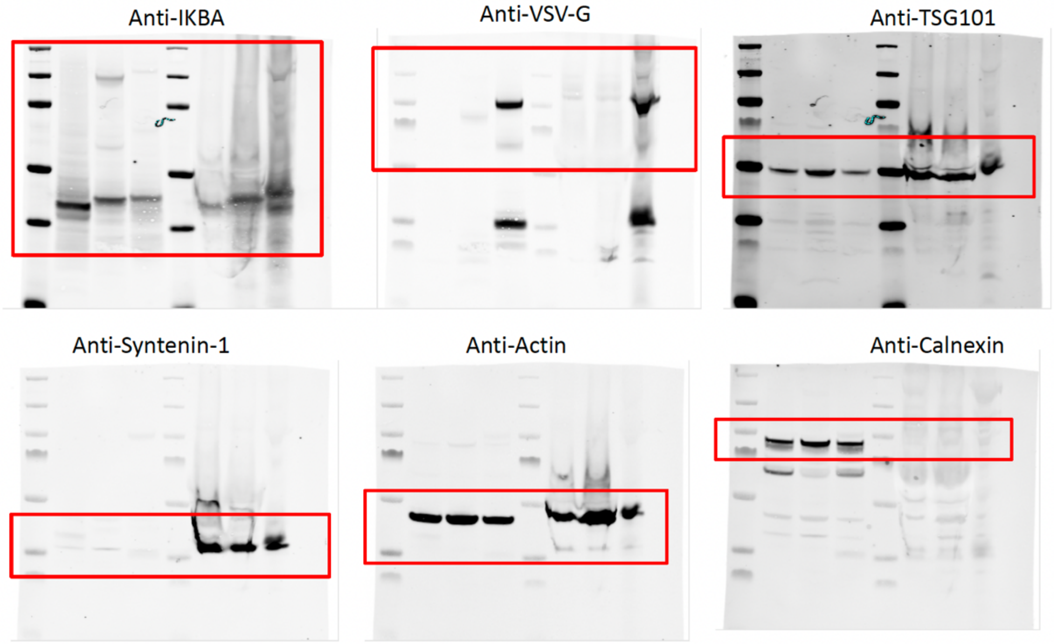

Fig. s16d: right panels

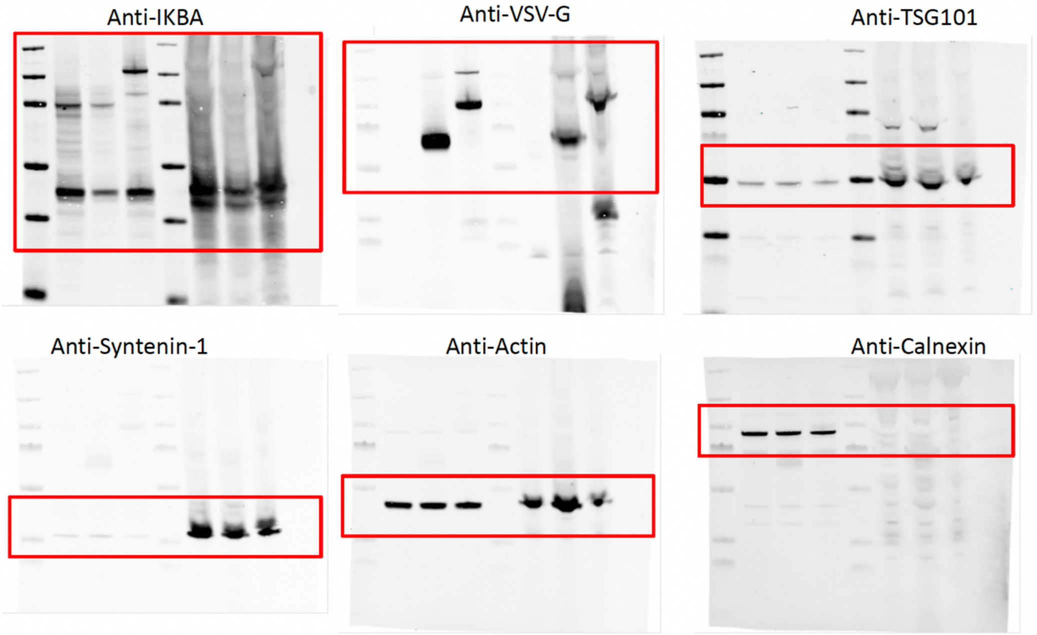

Supplement: Supplementary file 1 — Supplementary Information [file 41467_2025_59377_MOESM1_ESM.pdf]
