# Supplementary material for: Engineering of extracellular vesicles for efficient intracellular delivery of multimodal therapeutics including genome editors
Source: Nat Commun. 2025 Apr 29;16:4028. doi: 10.1038/s41467-025-59377-y (PMC12041237; doi:10.1038/s41467-025-59377-y)
Supplement: Supplementary file 2 — Description of Additional Supplementary Files [file 41467_2025_59377_MOESM2_ESM.pdf]

Description of Additional Supplementary Files

File Name: Supplementary Movie 1
Description: Dynamic GFP expression in HeLa-TL cells treated with VEDIC (VSV-G+CD63-Intein-Cre) EVs.

File Name: Supplementary Movie 2
Description: Dynamic GFP expression in HeLa-TL cells treated with control (CD63-Intein-Cre) EVs.

File Name: Supplementary Movie 3
Description: Dynamic GFP expression in HeLa-TL cells treated with VFIC (VSV-G-Foldon-Intein-Cre) EVs.
