# Supplementary material for: Engineering of extracellular vesicles for efficient intracellular delivery of multimodal therapeutics including genome editors
Source: Nat Commun. 2025 Apr 29;16:4028. doi: 10.1038/s41467-025-59377-y (PMC12041237; doi:10.1038/s41467-025-59377-y)
Supplement: Supplementary file 6 — Reporting Summary [file 41467_2025_59377_MOESM6_ESM.pdf]

nature portfolio

Corresponding author(s): Xiuming Liang
Samir EL Andaloussi
Joel Nordin

Last updated by author(s): Mar 17, 2025

Reporting Summary

Nature Portfolio wishes to improve the reproducibility of the work that we publish. This form provides structure for consistency and transparency in reporting. For further information on Nature Portfolio policies, see our Editorial Policies and the Editorial Policy Checklist.

Statistics

For all statistical analyses, confirm that the following items are present in the figure legend, table legend, main text, or Methods section.

n/a	Confirmed	
☐	☒	The exact sample size (*n*) for each experimental group/condition, given as a discrete number and unit of measurement
☐	☒	A statement on whether measurements were taken from distinct samples or whether the same sample was measured repeatedly
☐	☒	The statistical test(s) used AND whether they are one- or two-sided<br>*Only common tests should be described solely by name; describe more complex techniques in the Methods section.*
☒	☐	A description of all covariates tested
☒	☐	A description of any assumptions or corrections, such as tests of normality and adjustment for multiple comparisons
☐	☒	A full description of the statistical parameters including central tendency (e.g. means) or other basic estimates (e.g. regression coefficient) AND variation (e.g. standard deviation) or associated estimates of uncertainty (e.g. confidence intervals)
☐	☒	For null hypothesis testing, the test statistic (e.g. F, t, r) with confidence intervals, effect sizes, degrees of freedom and P value noted<br>*Give P values as exact values whenever suitable.*
☒	☐	For Bayesian analysis, information on the choice of priors and Markov chain Monte Carlo settings
☒	☐	For hierarchical and complex designs, identification of the appropriate level for tests and full reporting of outcomes
☒	☐	Estimates of effect sizes (e.g. Cohen's *d*, Pearson's *r*), indicating how they were calculated

Our web collection on statistics for biologists contains articles on many of the points above.

Software and code

Policy information about availability of computer code

Data collection	Microsoft excel, NanoSight NTA software, MACSQuant Analyzer 10 flow cytometer, SoftMax Pro, Amnis ImageStream X Mk II and Amnis Cellstream software. Nanoimager S Mark II microscope software.
Data analysis	Prism 9, Microsoft excel, FlowJo R (version 4.4.2) for statistics;Flow cytometry: FlowJo (v.10.6.2); Size distribution and particle concentration: NTA analytical software (v. 3.2)

For manuscripts utilizing custom algorithms or software that are central to the research but not yet described in published literature, software must be made available to editors and reviewers. We strongly encourage code deposition in a community repository (e.g. GitHub). See the Nature Portfolio guidelines for submitting code & software for further information.

Data

Policy information about availability of data

All manuscripts must include a data availability statement. This statement should provide the following information, where applicable:

- Accession codes, unique identifiers, or web links for publicly available datasets
- A description of any restrictions on data availability
- For clinical datasets or third party data, please ensure that the statement adheres to our policy

Source data are provided as a Source Data file.

Research involving human participants, their data, or biological material

Policy information about studies with human participants or human data. See also policy information about sex, gender (identity/presentation), and sexual orientation and race, ethnicity and racism.

Reporting on sex and gender	Not applicable.
Reporting on race, ethnicity, or other socially relevant groupings	Not applicable.
Population characteristics	Not applicable.
Recruitment	Not applicable.
Ethics oversight	Not applicable.

Note that full information on the approval of the study protocol must also be provided in the manuscript.

Field-specific reporting

Please select the one below that is the best fit for your research. If you are not sure, read the appropriate sections before making your selection.

☒ Life sciences ☐ Behavioural & social sciences ☐ Ecological, evolutionary & environmental sciences

For a reference copy of the document with all sections, see nature.com/documents/nr-reporting-summary-flat.pdf

Life sciences study design

All studies must disclose on these points even when the disclosure is negative.

Sample size	The sample size were determined without any pre-calculations of the number needed, instead the sample size was based on educated assumptions based on previous experience of the experimental set up of the in vitro and in vivo experiments.
Data exclusions	No data were excluded
Replication	In vitro work were done with biological replicates. Animals used in each experiment range from 3 to 10 per group.
Randomization	division into the different experimental groups were conducted in each experiment, after cells had been plated or mice had been inoculated, by randomly choosing wells or mice, respectively, for each treatment group.
Blinding	No blinding of the in vitro or in vivo work was conducted. All in vivo experiments were conducted together with at least two investigators and the measurements were divided between the researchers on different days in to minimize bias.

Reporting for specific materials, systems and methods

We require information from authors about some types of materials, experimental systems and methods used in many studies. Here, indicate whether each material, system or method listed is relevant to your study. If you are not sure if a list item applies to your research, read the appropriate section before selecting a response.

Materials & experimental systems

n/a	Involved in the study	
☐	☒	Antibodies
☐	☒	Eukaryotic cell lines
☒	☐	Palaeontology and archaeology
☐	☒	Animals and other organisms
☒	☐	Clinical data
☒	☐	Dual use research of concern
☒	☐	Plants

Methods

n/a	Involved in the study	
☒	☐	ChIP-seq
☐	☒	Flow cytometry
☒	☐	MRI-based neuroimaging

Antibodies

Antibodies used	For the dilution of primary antibodies, 1:20000 for Actin and 1:1000 for all other antibodies. For dilution of secondary antibodies, 1:10000 was used. The following is the detailed information about the antibodies used in this study.

	Primary antibodies: Rat anti-NeuN (Cat#: MAB377, Millipore), Rat anti-Mouse CD8a (Cat#: 553027, BD Pharmingen), Rabbit anti-GFP (Cat#: ab290, Abcam), rb-α-ms IBA1 (Cat#: 019-1941, Wake), rt-α-ms GFAP (Cat#: ab53554, Abcam), rt-α-ms RFP (Cat#: 600-401-379-RTU,Thermo Fisher Scientific), rt-α-ms CD45 (Cat#: ab10558, Abcam), rt-α-ms B220 (Cat#: 14-0452-82, Thermo Fisher Scientific), rt-α-ms F4/80 (Cat#: MCA497G, Bio-Rad), anti-CD63 (ab134045, Abcam), anti-Alix (MA1-83977, Thermofisher), Anti-Cre (Cat#: ab188568, Abcam), anti-Tsg101 (ab30871, Abcam), Goat anti-VSV-G (Cat#: PA1-30278, Thermo Fisher Scientific), Mouse anti-Cas9 (Cat#: MA5-23519, Thermo Fisher Scientific), Goat anti-Calnexin (Cat#: MCA497G, Thermo Fisher Scientific), Mouse anti-β-Actin (Cat#: A5441, Sigma), Mouse anti- IkB-alpha (Cat#: MA5-15132, Thermo Fisher Scientific), Rabbit anti-PCSK9 (Cat#: PA5-78663, Thermo Fisher Scientific),Rabbit anti-Syntenin-1 (Cat#: 600-401-379-RTU, Thermo Fisher Scientific);<br>Secondary antibodies: Alexa Flour 488 goat anti-mouse (Cat#: A11001,Thermo Fisher Scientific), Alexa Fluor 633 goat anti-rabbit (Cat#: A21070, Thermo Fisher Scientific), Alexa Fluor 568 goat anti-rat (Cat#: A11077, Thermo Fisher Scientific), Alexa Fluor 568-conjugated streptavidin (Cat#: S11226, Thermo Fisher Scientific), Alexa Fluor 488 goat anti-rat (Cat#: ab150077, Abcam), IRDye® 800CW Donkey anti-Goat IgG (Cat#: 926-32214, LI-COR Biosciences), IRDye® 800CW Goat anti-Mouse IgG (Cat#: 926-32210, LI-COR Biosciences), IRDye® 800CW Goat anti-Rabbit IgG (Cat#: 926-32211, LI-COR Biosciences), IRDye® 680RD Goat anti-Mouse IgG (Cat#: 926-68070, LI-COR Biosciences), IRDye® 800CW Donkey anti-Goat IgG (Cat#: 926-32214, LI-COR Biosciences).
Validation	Validation of the antibodies had been conducted by the distributor and was not further validated other than as described in the manuscript.

Eukaryotic cell lines

Policy information about cell lines and Sex and Gender in Research

Cell line source(s)	HEK293T(ATCC, CRL-3216), Huh7 (XenoTech, JCRB0403) , MSC (ATCC, PCS-500-010), HeLa,Raw264.7, THP-1, K562, T47D, and B16F10 cells were all either bought in or given as a kind gift from collaborators.
Authentication	Non of these cell lines were authenticated other then by morphology
Mycoplasma contamination	All cell lines were confirmed negative by testing for mycoplasma and the mycoplasma test was performed regularly in the lab.
Commonly misidentified lines<br>(See ICLAC register)	no misidentified lines were used

Animals and other research organisms

Policy information about studies involving animals; ARRIVE guidelines recommended for reporting animal research, and Sex and Gender in Research

Laboratory animals	Mouse, female or male, C57BL/6, weight 18-22g and Cre-LoxP tdTomato mice, 18-22g, housed in our animal facility for at least one week before use according to standard routines (temperature: 20-22°C, humidity: 45-55%, dark/light cycle: 12/12 h).
Wild animals	The study did not involve wild animals
Reporting on sex	only female mice were used in order to reduce the number of mice and unwanted factors, owing to the fact that male mice may fight more with each other for the intratumoral model and LPS model; both female and male mice were used for the Cre delivery experiment because the breeding capacity of these mice will determine the gender balanced design of using the B6.Cg-Gt(ROSA)26Sortm9(CAG-tdTomato)Hze/J (Rosa26.tdTomato)) reporter mice.
Field-collected samples	All mice were kept at a dedicated laboratory animal facility with controlled temperature, light-dark cycles, with access to nesting material, food and water.
Ethics oversight	All mouse experiments were performed in accordance with the ethical permission granted by The Swedish Local Board for Laboratory Animals and designed to minimize the suffering and pain of the animals, with ethical permit number (LPS model:16212-2020; tumor model:2173-2021); or approved by Inflammation Research Center, Ghent University with ethical permit number: LA1400091/ LA2400526.

Note that full information on the approval of the study protocol must also be provided in the manuscript.

Flow Cytometry

Plots

Confirm that:

☒ The axis labels state the marker and fluorochrome used (e.g. CD4-FITC).

☒ The axis scales are clearly visible. Include numbers along axes only for bottom left plot of group (a 'group' is an analysis of identical markers).

☒ All plots are contour plots with outliers or pseudocolor plots.

☒ A numerical value for number of cells or percentage (with statistics) is provided.

Methodology

Sample preparation	Sample preparations for either cells or EV measurements by flow cytometry are detailed in Methods.
Instrument	MACSQuant Analyzer 10 flow cytometer and Amnis Cellstream.
Software	The respective instrument's software were used to acquire the data. Flow Jo was used for analysis.
Cell population abundance	For cells: the percentage of parent population and mean fluorescence intensity of single alive cells<br>For EVs: concentration oand mean fluorescence intensity f fluorescent particles
Gating strategy	The gating strategies are shown in the supplementary information. For cells, single (FSC-A vs FSC-H) > cells > alive (DAPI-negative) > mNG;<br>For EVs: SSC-low > Time > mNG/APC (refer to our previous publication: doi.org/10.1002/jev2.12238).

☒ Tick this box to confirm that a figure exemplifying the gating strategy is provided in the Supplementary Information.
